# Inorganic Nanomaterials can Promote or Inhibit Tumor Metastasis Depending on Tumor Microenvironment Composition

**DOI:** 10.1002/advs.77310

**Published:** 2026-08-20

**Authors:** Kiana Buttiens, Xiaoqi Zheng, Xueqi Fan, Mukaddes Izci, Christy Maksoudian, Irati Perez Gilabert, Tianjiao Chu, Filipa Goncalves, Jonas Dehairs, Johan Swinnen, Bella B. Manshian, Stefaan J. Soenen

**Affiliations:** ^1^ NanoHealth and Optical Imaging Group Department of Imaging and Pathology KU Leuven Leuven Belgium; ^2^ Leuven Cancer Institute KU Leuven Leuven Belgium; ^3^ Laboratory of Lipid Metabolism and Cancer Department of Oncology KU Leuven Leuven Belgium; ^4^ Translational Cell and Tissue Research Unit Department of Imaging and Pathology KU Leuven Leuven Belgium

**Keywords:** metastasis, nanomaterials, oncology

## Abstract

The growing application of engineered nanomaterials (NMs) in cancer imaging and therapy highlights the need to better understand their effects on tumors and the tumor microenvironment (TME). Previous studies have reported conflicting findings, suggesting that NMs can either enhance or suppress tumor metastasis. In this study, we investigated the impact of gold (Au) and silica‐coated gold (Au_Sil_) nanomaterials on metastatic progression in two triple‐negative breast cancer (TNBC) models. Despite having similar size and surface characteristics, the NMs produced distinct, tumor‐specific effects, resulting in either increased or decreased metastatic dissemination depending on the experimental context. Further analysis of the TME revealed several factors influencing these outcomes. Both Au and Au_Sil_ NMs increased endothelial permeability, while Au NMs also stimulated cancer cells to produce anti‐angiogenic factors. In both cases, higher levels of hypoxic tumor‐associated macrophages were associated with reduced endothelial vessel maturity. Importantly, NM‐induced increases in metastasis could be mitigated through pharmacological vessel normalization or inhibition of hypoxia‐related signaling pathways. Overall, these findings demonstrate that Au and Au_Sil_ nanomaterials exert context‐dependent effects on metastasis by modifying the TME, while also identifying strategies to improve their safe and effective use in cancer applications.

## Graphical abstract

As engineered nanomaterials gain prominence in cancer imaging and therapy, understanding their impact on tumor progression becomes increasingly critical. We demonstrate that gold and silica‐coated gold nanomaterials can either suppress or enhance breast cancer metastasis through distinct tumor microenvironment interactions, while identifying mechanistic pathways and therapeutic strategies that improve their safety and efficacy.

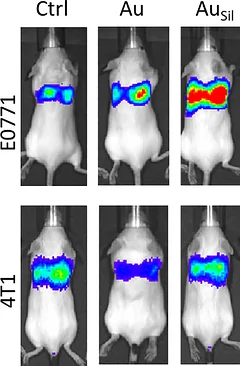

## 1 Introduction

Nanomaterials (NMs) have been widely studied as contrast or therapeutic agents for nearly two decades [1, 2, 3, 4]. Inorganic NMs have primarily served as contrast agents, but are also being explored more for therapy, in particular in the field of oncology [5, 6, 7], drug delivery, and tissue related pathologies [8, 9, 10]. Gold nanoparticles (Au NPs) are widely applied for their biocompatibility, stability, and tuneability. Due to their unique characteristics, largely derived from its localized surface plasmon resonance (LSPR), they are of use for diagnostic as well as therapeutic purposes, such as photodynamic imaging or thermal ablation of solid tumors [11]. Another widely used NP type within the field of oncology is silica, as it is also biocompatible, stable, and easy to modify, making it a highly suitable NP type for diagnostic or delivery platforms in multiple pathologies [12].

One of the main research topics has been to improve the delivery of systemically administered NMs to the tumor site [13]. Classically, these NMs will be rapidly cleared by cells of the reticuloendothelial systems (RES), resulting in only a very minor proportion of these NMs to reach the actual tumor [14]. Some improvements have been suggested, such as the use of peptides (*e.g*. cyclic iRGD) to promote transcytosis across tumor blood vessels or an in‐depth characterization of tumor physiology with a focus on leakiness of tumor‐associated blood vessels (the extravasation potential of NMs) [15]. However, no major breakthroughs have been reported thus far that can unambiguously promote NM delivery to solid tumors.

The process of NM delivery to solid tumors was long considered to be caused by the so‐called enhanced permeability and retention (EPR) effect [16]. In short, this refers to the passivation of the NM surface to increase blood circulation times by preventing opsonization and premature RES‐mediated clearance, enabling the NMs to pass the tumor‐associated blood vessels multiple times, where they would be able to extravasate by passing through gaps in the blood vessel walls [17]. In tumors, newly formed blood vessels can often be immature and not yet have a perfect endothelial layer, displaying several gaps between endothelial cells. The process of NM delivery via EPR has been widely reported and is described as highly ineffective, as NM transport into the tumor mass is impeded by the high interstitial pressure in the tumor and a dense extracellular matrix (ECM) network that typically accompanies tumors [18]. This is illustrated by the extremely low median NM accumulation (0.7%) at the tumor site upon passive delivery [19], which has been confirmed by earlier studies from our end for gold NPs specifically [20], The process of EPR has also recently been called into question. Works by the group of Warren Chan and colleagues found that NM delivery across the tumor‐associated blood vessels occurred via an active transport mechanism, and not a passive one such as EPR [21]. In a follow‐up study, they found that transport of NMs was mediated by specialized tumor‐associated endothelial cells (TECs) [22]. Other studies from Peng et al., found that NMs such as gold nanoparticles (NPs) are able to generate gaps between endothelial cells themselves [23]. The systemic delivery of NMs therefore results in a close interaction of the NPs with the tumor microenvironment such as the TECs. The study by Peng and colleagues observed that the NM‐mediated endothelial leakiness furthermore had profound effects on tumor malignancy by promoting tumor cell intravasation through the newly formed gaps and thus promoting metastasis [23]. Other studies however have reported that the same type of gold NMs were able to reduce metastasis formation upon systemic delivery. This has been linked with a normalization of tumor blood vessels and reduction of epithelial to mesenchymal transition (EMT) [24], inhibition of MAPK signaling and reversal of EMT in ovarian tumors [25], or the reversal of cancer‐associated fibroblast (CAF) activation [26, 27].

The apparently contradictory effects of inorganic NMs on metastasis suggest that their biological consequences cannot be explained by endothelial leakiness alone. Instead, metastatic outcome is likely determined by the interaction between NM‐induced changes in vascular barrier function, the inflammatory and hypoxic state of the TME, and the intrinsic response of tumor cells to NM exposure.

In the present study, we used size‐matched Au and Au_Sil_ NMs in two orthotopic triple‐negative breast cancer models to dissect these context‐dependent effects. Rather than proposing a single linear pathway, we aimed to identify which TME components functionally determine whether NM exposure promotes or suppresses metastasis. We therefore examined direct NM effects on tumor endothelial permeability, indirect effects mediated by tumor cells and stromal cells, the contribution of tumor‐associated macrophage (TAM) abundance and hypoxia‐associated REDD1 signaling, and the potential of vessel normalization or hypoxia inhibition to mitigate NM‐associated metastatic risk. This approach allowed us to distinguish associative stromal changes from functionally supported determinants of NM‐driven metastatic outcome.

## 2 Materials and Methods

### 2.1 Nanomaterials and Nanomaterial Characterization

Gold and SiO_2_‐coated Au NMs were purchased from NanoComposix. The Au NMs were 60 nm diameter, while Au_Sil_ NMs had a 20 nm Au core surrounded by a 20 nm thick SiO_2_ layer, resulting in a total diameter of 60 nm as well. Both NMs were further coated with polymethacrylic acid (PMA) which was further derivatized with poly(ethylene glycol) (PEG), with a methoxyl end group and of 5 kDa chain length. The NMs were provided in water at 1 mg/mL concentration. The NMs were characterized using Transmission electron microscope (TEM) to determine their shape and size, Dynamic light scattering (DLS) to measure hydrodynamic diameter and surface charge, and electrophoretic mobility measurements for ζ‐potential analysis.

### 2.2 TEM

Formvar film coated 400‐mesh copper grids (Agar Scientific Ltd., England) were used. The grids were first glow‐discharged to improve NM adsorption efficacy. Diluted NM suspensions (25 µL) were dropped on the grids and left to evaporate. Finally, images were acquired using a JEM‐1400 transmission electron microscope (JEOL, Japan) at an accelerating voltage of 80 keV.

### 2.3 DLS

Dynamic light scattering (Malvern Panalytical, Zetasizer Nano ZSP, United Kingdom) was applied to define hydrodynamic size distribution and polydispersity index (PDI), while the same device was also used to perform electrophoretic mobility measurements to obtain the ζ‐potential. The NMs were diluted in PBS. Measurements were performed at 25°C, registering light scattering intensity at an angle of 90°. For each sample, an average of 3 measurement series was established.

### 2.4 Cell Culture

TNBC cell lines 4T1 and E0771 were used throughout this study. 4T1 were cultured in Dulbecco's Modified Eagles Medium (DMEM), supplemented with 10% heat‐inactivated fetal bovine serum (FBS), while E0771 cells were maintained in RPMI medium with 10% FBS. The media and supplements were purchased from Gibco (Invitrogen, Belgium). All cell types were maintained in a humidified atmosphere at 37°C and 5% CO_2_.

### 2.5 Animal Experiments

Female Balb/c and C57Bl6 mice (Harlan Laboratories, Cambridgeshire, UK), 5–7 weeks old, were used in this study. The animal studies used orthotopic syngeneic tumor models in which Balb/c or C57Bl6 animals received 500,000 4T1 or E0771 cells, respectively, in saline as an injection into the 4^th^ mammary fat pad. All mouse surgical procedures and imaging were performed with the animals anesthetized by inhalation of 2% isoflurane. The condition of the animals was monitored every day, and their weight was measured every other day. Tumors were measured with calipers every other day. When tumors reached the size of minimally 50 mm^3^ (approximately 10–14 days after tumor inoculation), the animals were divided into different groups of similar tumor size for further experiments. For euthanasia, animals were subjected to 5% isoflurane inhalation. To ensure death following isoflurane inhalation, cervical dislocation was performed. Tumors were then removed from the animals and used for *ex vivo* analysis or for tissue dissociation to isolate cell populations of interest. All animal studies were approved by KU Leuven's Institutional Animal Care and Use Committee (IACUC; approval number P149/2019) in accordance with the principles and procedures outlined in national and European regulations.

### 2.6 NM Administration

When orthotopic E0771 or 4T1 tumors had reached about 50 mm^3^ in size, animals received a single bolus administration of 100 µg of NMs by intravenous injection. The animals were then left, and tumor sizes were measured every other day. After a total of 25 days (18 days post NM administration), the animals were sacrificed, and the tumor, lungs, and blood were collected for *ex vivo* analysis.

### 2.7 Cell Isolation and Ex Vivo Culture

Mouse tumor tissue was dissociated into a single cell suspension using the Tumor Dissociation Kit, mouse in combination with the gentleMACS Octo Dissociator (Miltenyi Biotec) according to the manufacturer's instructions. A custom‐made protocol was run on the gentleMACS Octo Dissociator. In brief, the tissue was incubated for 1 h at 37°C and under constant rotation at 20 rpm, according to company protocol.

CAFs were isolated by magnetic activated cell sorting (MACS) based on CD90.2 expression. After tumor dissociation fibroblasts were first pre‐enriched using flow cytometry, following staining against CD45 lymphocytes and GFP^+^ tumor cells, where only the negative cells were kept and this negative population was then used to isolate CAFs using the Tumor‐associated Fibroblast Isolation Kit, mouse (Miltenyi Biotec) with LD‐ and MS columns (Miltenyi Biotec), respectively. The isolation procedure was performed according to the manufacturer's instructions.

TAMs were isolated from the dissociated tumors by pre‐enrichment using CD45 microbeads and LD columns for negative selection (Miltenyi Biotec), and purified using the EasySep Mouse F4/80 Positive Selection Kit (Stem Cell Technologies) using positive selection.

TECs were isolated from dissociated tumors by pre‐enrichment using CD45 microbeads and LD columns for negative selection (Miltenyi Biotec), and purified using CD31 microbeads (Miltenyi Biotech) using positive selection.

Purity and yield of all cell types were assessed by flow cytometric analysis using fluorochrome‐conjugated CD45 antibodies (negative selection), CD90.2 antibodies (CAF), F4/80 (TAM), and TECs (CD31).

All isolated primary cells were plated on Matrigel (Corning) coated tissue culture dishes and grown in DMEM supplemented with 8% FCS, 10 mM HEPES, 2 mM L‐glutamine, and 1x non‐essential amino acids (NEAA, Gibco, Invitrogen, Belgium) at 37°C in a humidified 5% CO_2_ atmosphere. Detailed experimental procedures are described in the supporting information.

## 3 Results and Discussion

### 3.1 Au and AuSil NMs Differentially Affect Metastatic Dissemination in 4T1 and E0771 Tumors

The Au NPs and Au_Sil_ NPs display similar physicochemical characteristics (Extended Data Figure S1), with good colloidal stability, core sizes of approximately 60 nm, and negative surface potentials. To evaluate the effect of the NMs on tumors, we opted to work on triple negative breast cancer (TNBC). TNBC is an aggressive subtype of breast carcinoma accounting for about 15% of diagnosed breast cancers worldwide. Recurrence and metastasis of TNBC is common, and the median overall survival for metastatic TNBC patients is only 12 months [28], highlighting an urgent need for improved therapies, in particular for those that could reduce metastases levels, as tumor cell dissemination can account for up to 90% of tumor mortality [29]. To study the metastasis levels of murine TNBC models, we have opted for 2 frequently used TNBC cell lines with documented occurrence of pulmonary metastasis, being 4T1 and E0771, and analysed their growth and level of pulmonary metastases in the presence or absence of the Au and Au_Sil_ NMs. To this end, 4T1 and E0771 TNBC cells were selected for in vivo analysis of NM biodistribution (Extended Data Figure S2) which revealed no clear differences between the two types of NMs, nor between the two tumor types. Interestingly, upon looking at the level of NM uptake in the tumor and tumor cells, specifically, this did not correlate with the level of endothelial leakiness (Extended Data Figure S3), and rather the opposite behavior seemed to occur, where those animals with higher levels of tumor‐specific NM uptake levels were in general those with lower endothelial permeability levels, and vice versa. These data, while being somewhat contradictory to the common concept of the EPR effect, are in line with previous reports where active transport of NMs across endothelial cells has been suggested as the main route of NM delivery to solid tumors [22, 30]. In terms of tumor‐related parameters (Extended Data Figure S3), an overall higher necrosis and ECM density impaired NM delivery, but higher levels of endothelial cell coverage, maturity of blood vessels, levels of TAMs and CAFs all promoted higher NM delivery levels. These effects are in line with the classical view of EPR, where necrotic regions are less perfused, and high ECM density prevents NP extravasation and transport of the NMs throughout the interstitial tissue. The influence of TAMs is also in line with expectations, where TAMs have been frequently described to take up NMs reaching the tumor site [31]. The influence of CAFs is somewhat more surprising, as higher CAFs levels are typically linked to higher ECM densities. However, as the nature and heterogeneity in CAFs functions are very broad and currently not fully understood [32], high levels of CAFs in the absence of increased ECM density has a positive effect on NM delivery. Another surprising finding is that while perfusion and vessel coverage in the tumor contribute to NM delivery, in support of EPR effects, vessel permeability did not play a particular role at any level, and mature vessels even contributed more to NP delivery. The latter may be due to the reduced interstitial pressure associated with mature vessels compare to leaky structures, which is known to enhance for example chemotherapy influx into tumors [33].

**FIGURE 1:**
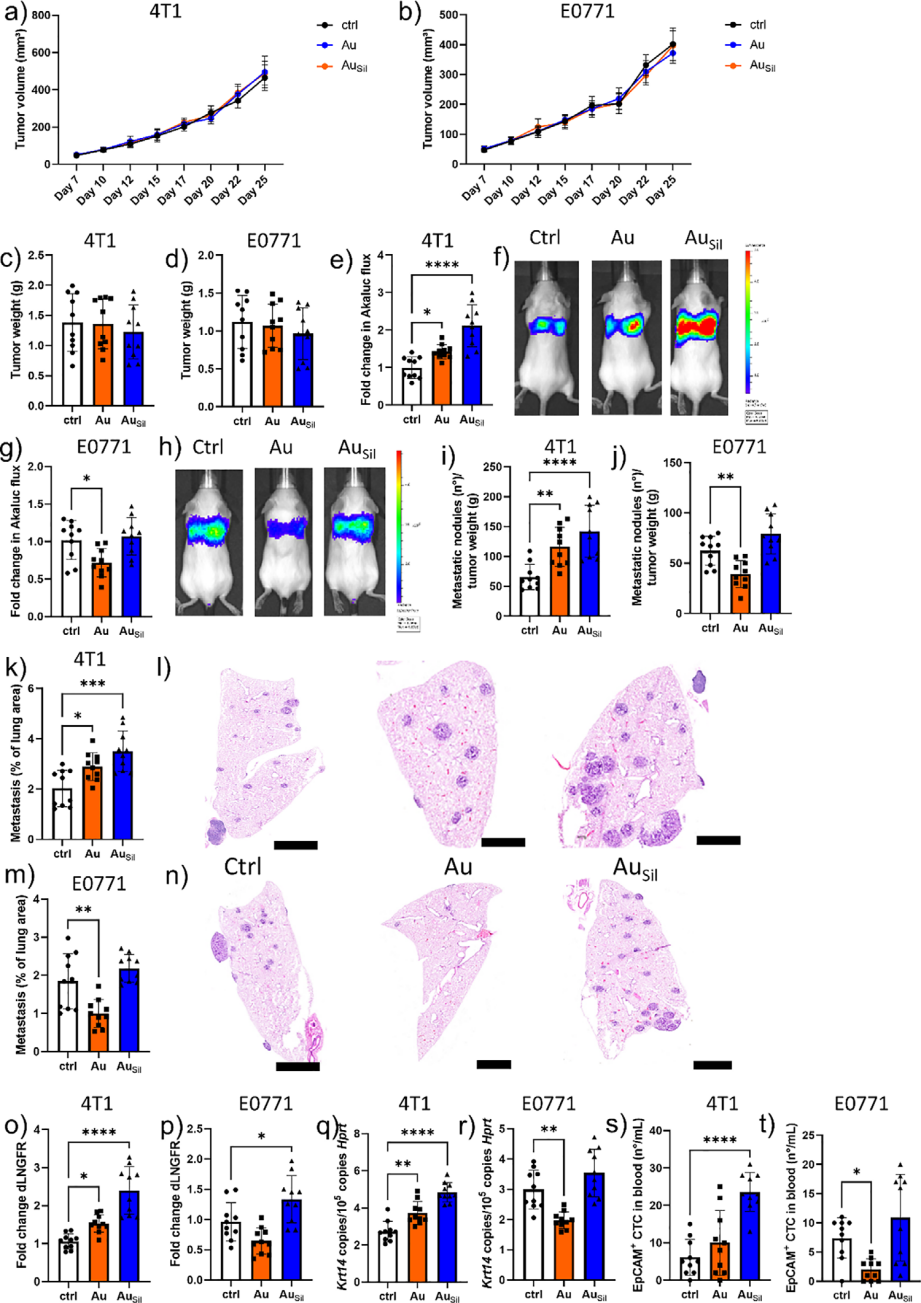
Au and Au_Sil_ NMs have profound effects on the level of metastasis depending on the NM and animal model used. a,b) Histograms expressing the tumor volume of untreated tumors (a: 4T1; b: E0771) or tumors in animals receiving 100 µg of Au or Au_Sil_ NMs as a function of time. The animals received the NMs at day 7 following tumor cell graft. c,d) Ex vivo weight of the tumors (c: 4T1; d: E0771) isolated 25 days after grafting, and 18 days following NM administration. e‐h) Akaluc luminescence signal measured over the lung area in tumor‐bearing mice (e: 4T1; g: E0771) 7 days following surgical removal of the tumor (32 days post initial tumor graft). Representative BLI images are shown for animals that had been originally grafted with (f: 4T1; h: E0771) tumors. Please note that for ease of imaging, albino mice were used for grafting E0771 cells. i,j) The number of metastatic nodules counted in the lungs for i) 4T1 and j) E0771 tumors at 25 days post grafting. k‐n) The area taken up by metastatic nodules (k: 4T1; m: E0771) in the lung tissue slice as determined by H&E staining relative to the area of the entire tissue slice. Representative H&E images of lungs obtained from animals originally grafted with (l: 4T1; n: E0771) tumors. Scale bars: 2 mm. o,p) Expression of the truncated dLNGFR receptor that was introduced into the tumor cells and determined by qPCR of lung tissue and expressed relative to the level of untreated tumor‐bearing mice (o: 4T1; p: E0771). q,r) The level of Krt14 expression relative to that of housekeeping gene Hprt determined by qPCR of blood samples taken at 25 days post tumor grafting (q: 4T1; r: E0771). s,t) The number of EpCAM^+^CD45^−^ cells detected in the blood at 25 days post tumor grafting expressed as the number of EpCAM^+^ cells per mL of sample and determined by ImageStream analysis (s: 4T1; t: E0771). Animals in the control group were administered PBS only. Significant differences between a treated group and untreated controls are indicated where relevant (p < 0.05: *, p < 0.01: **; p < 0.001: ***; p < 0.0001: ****) based on ANOVA testing using GraphPad Prism 9 (n = 8); data is represented as mean ± SD.

Delivery of either the Au or Au_Sil_ NMs to the 4T1 or E0771 tumors did not affect tumor growth at all (Figure 1a–d), however, longitudinal in vivo optical imaging hinted toward a difference in metastatic behavior toward the lungs between both types of NMs and both tumor types (Figure 1e–j). This was confirmed by H&E staining of lung tissues sections, displaying elevated lung metastases in the 4T1 model for both NM types (Figure 1k–n). Literature also supports this: 4T1 is a classic highly metastatic murine mammary carcinoma model that can spontaneously metastasize to multiple organs [34]; while E0771 originates from C57BL/6 spontaneous mammary tumour, and its phenotype and metastatic behavior are more heterogenous and its characterization is more complex [35]. In the E0771 model, the Au_Sil_ NMs had no significant effects, while the Au NMs significantly reduced the level of metastasis. To further support this finding, transgenic murine cell lines were used, expressing a qPCR‐traceable truncated low‐affinity nerve growth factor receptor (dLNGFR) [36], which is normally restricted to the central nervous system and therefore absent in the lungs. The qPCR data of lung tissues found the same trend as observed for the longitudinal in vivo optical imaging and H&E (Figure 1o,p). To examine whether the observed metastatic increase resulted directly from elevated cancer cell extravasation, blood samples were collected 48h following NM administration. The level of circulating tumor cells, examined by the number of *Krt14* ^+^ cells (a prognostic tumor marker in epithelial cancer types, which is known not to be naturally present in murine blood cells) [36] was elevated by both NMs in 4T1 bearing mice, while this was reduced in E0771 bearing mice exposed to Au NMs (Figure 1q,r). This was further confirmed by ImageStream analysis of Epcam^+^ (a diagnostic marker for various cancers) in lymphocyte‐depleted blood (Figure 1s,t). Together, the data indicated that in the 4T1 model, both NMs augmented the level of metastasis, but this was far more pronounced for Au_Sil_ NMs. These data were quite surprising, but also explained, in part, the apparent discrepancies that have been reported in literature. These findings indicate that the divergent responses of 4T1 and E0771 tumors are not explained by tumor growth or total NM delivery alone. We therefore next examined whether baseline differences in the TME could provide a permissive context for NM‐associated metastasis. Recent studies suggested that intravenously administered NMs could induce endothelial leakiness by interfering with VE‐cadherin‐mediated tight junctions and hereby promote tumor cell extravasation [23]. Other studies have suggested that NMs can impede metastasis, through a variety of signaling cascades, mainly involving CAFs [37]. Together, these data show that Au and Au_Sil_ NMs can produce distinct metastatic outcomes despite comparable nominal size, surface coating, and biodistribution. This suggests that the biological differences are not driven by gross delivery differences alone but are associated with the different inorganic architectures of the two formulations. Because the current design does not isolate a single material parameter, we next focused on identifying the biological responses that distinguish Au from Au_Sil_ exposure under otherwise comparable size and coating conditions.

### 3.2 NMs can Impact TEC Permeability, but This is Countered by Tumor Cell Signaling in a NM‐Specific Manner

**FIGURE 2:**
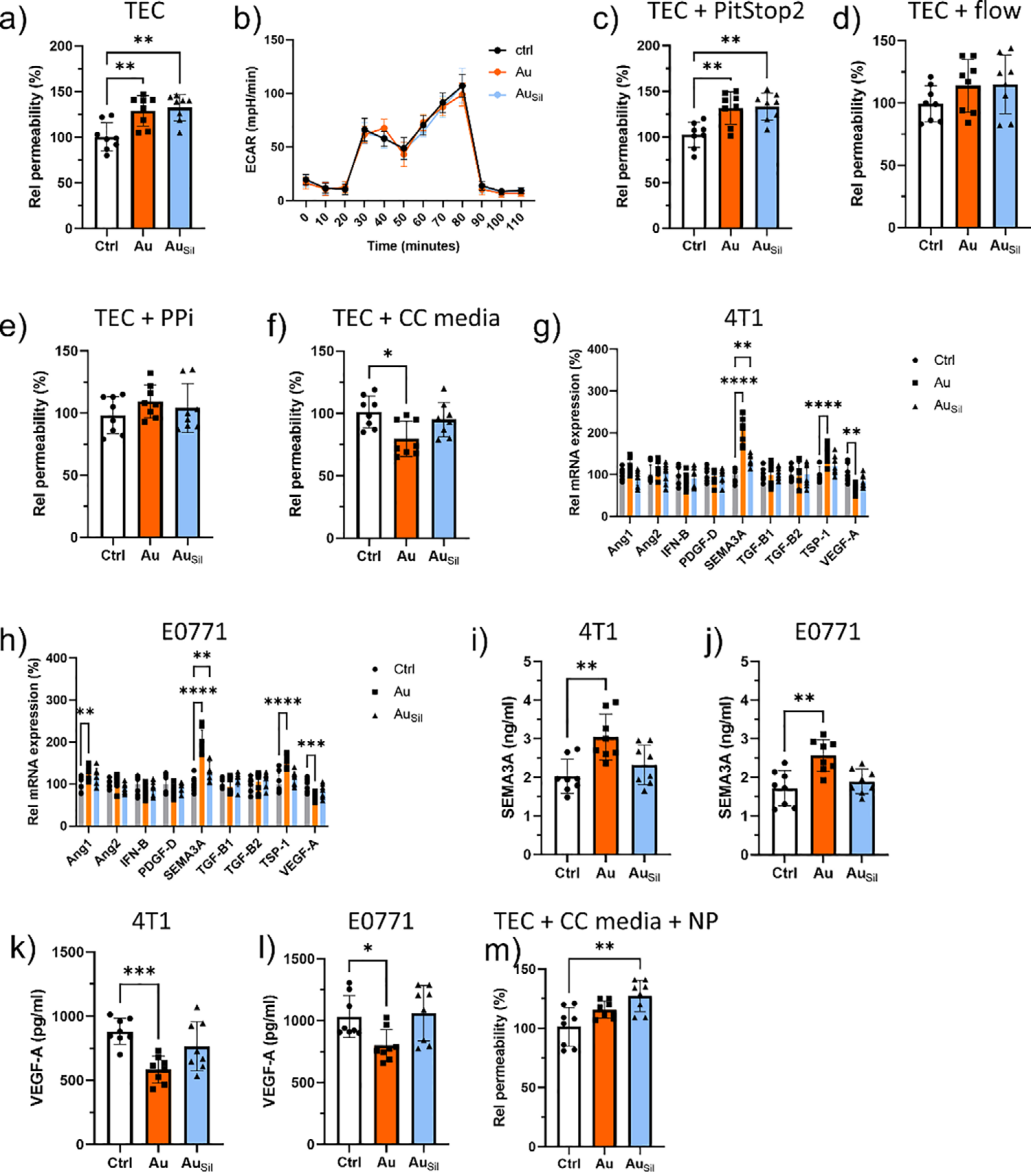
Au and AuSil NM increase endothelial permeability, but Au NMs also trigger cancer cell excretion of antiangiogenic factors to partially counter this effect. a) Histograms displaying the relative permeability of TEC monolayers when treated with Au or Au_Sil_ NMs. b) Graph displaying the extracellular acidification rate of TECs when treated with Au or Au_Sil_ NMs. c) Histograms displaying the relative permeability of TEC monolayers when treated with Au or Au_Sil_ NMs in the presence of endocytosis inhibitor PitStop2. d) Histograms displaying the relative permeability of TEC monolayers when treated with Au or Au_Sil_ NMs when cells were exposed to the NMs in a flow rather than a static incubation. e) Histograms displaying the relative permeability of TEC monolayers when treated with Au or Au_Sil_ NMs in the presence of PP inhibitors (PPi) which are known to inhibit VE‐cadherin signaling. f) Histograms displaying the relative permeability of TEC monolayers when treated with tumor cell conditioned media obtained from 4T1 cells previously exposed to Au or Au_Sil_ NMs. g,h) Histograms indicating mRNA expression of genes involved in angiogenesis and vessel maturation assessed in (g: 4T1; h: E0771) tumor cells exposed to Au or Au_Sil_ NMs. i,j) Histograms displaying the level of secreted SEMA3A by (i: 4T1; J: E0771) tumor cells incubated with Au or Au_Sil_ NMs. k,l) Histograms displaying the level of secreted VEGF‐A by (k: 4T1; l: E0771) tumor cells incubated with Au or Au_Sil_ NMs. m) Histograms displaying the relative permeability of TEC monolayers when treated with tumor cell conditioned media obtained from 4T1 cells previously exposed to Au or Au_Sil_ NMs in the presence of Au or Au_Sil_ NMs. Control treatment groups received growth medium in the absence of NMs. Significant differences between a treated group and untreated controls are indicated where relevant (p < 0.05: *, p < 0.01: **; p < 0.001: ***; p < 0.0001: ****) based on ANOVA testing using GraphPad Prism 9 (n = 8); data is represented as mean ± SD.

Primary CAFs, TAMs or TECs were isolated and analyzed in view of their role in the observed differences. First, we looked at TECs, as they have been closely linked with tumor cell extravasation. Viability of isolated TECs was not affected by the Au or Au_Sil_ NMs under the conditions used (Extended Data Figure S4a), while permeability of TEC monolayers for fluorescently‐tagged dextran was significantly elevated by both NM types (Figure 2a). Neither of the NMs was found to have an impact on TEC metabolism as displayed by their similar extracellular acidification rates (ECAR; Figure 2b), which resulted in no difference in levels of glycolysis, glycolytic capacity of reserve (Extended Data Figure S4b–d). Using a general endocytosis inhibitor, PitStop2, NM uptake was found to be significantly impeded (Extended Data Figure S4e), without any impact on TEC permeability (Figure 2c). These data suggest that permutations in TEC monolayer integrity are caused by NM in the media, rather than internalized NMs. The latter data are in line with the reports by Peng et al., whom described the creation of NM‐induced junctions between endothelial cells resulting in elevated tumor cell extravasation [23]. When NMs were administered to TEC monolayers under continuous flow conditions mimicking the blood circulation, the impact on permeability was reduced (Figure 2d). As flow conditions will reduce the possible settlement of NMs on top of the TECs, this may indicate that close interactions of the NMs and the TECs are required to cause TEC monolayer leakiness. To confirm the occurrence of NM‐induced endothelial leakiness, the involvement of VE‐cadherin signaling, shown to be essential for this process, was evaluated by exposed the cells with Src kinase inhibitor PP1, which blocks VE‐cadherin signaling [38]. This was found to completely block NM‐induced TEC permeability (Figure 2e). Gene expression studies on isolated TECs found no significantly altered genes involved in tumor cell motility or epithelial to mesenchymal transition (EMT) levels (Extended Data Figure S4f,g). Incubation of TECs with conditioned media obtained from tumor cells exposed previously to the NMs, revealed that Au NMs significantly reduced TEC permeability (Figure 2f). The effect of Au NMs on cancer cells opposes the enhanced permeability upon direct interaction with TECs. To look into the potential mechanisms, gene expression in 4T1 and E0771 cells indicated enhanced levels of *Sema3A*, *Tsp‐1*, and reduced *Vegf‐a* levels (Figure 2g,h). This was confirmed by ELISAs showing increase SEMA3A and reduced VEGFA secretion by both cell types upon Au NM exposure (Figure 2i–l). These findings are in line with literature, where Au NMs have been described to increase SEMA3A [39]. Furthermore, VEGFA reduction and SEMA3A are markers for reduced angiogenesis and increased tumor vessel normalization, which impedes metastasis [40]. When exposing TEC monolayers to conditioned media in the presence of the NMs, Au NMs were found not to have an effect, while Au_Sil_ NMs caused a significant increase in permeability (Figure 2m). The results in Figure 2m seem to align with the results obtained in Figure 2a and Figure 2f. While in Figure 2a, both NM types result in elevated TEC permeability, Figure 2f indicates that in the absence of NMs, cancer cells previously exposed to the NMs can have differential effects. Au NMs appear to mediate the secretion of cancer cell‐derived factors that reduce TEC permeability, whereas Au_Sil_ NMs do not appear to affect cancer cells in the same way. When TEC permeability was then measured in the presence of the NMs and of conditioned media of cancer cells previously exposed to the NMs, the Au_Sil_ NMs still significantly increase TEC permeability, as the Au_Sil_ NM do this on their own and the conditioned media had no noticeable effect. However, Au NMs no longer have a significant effect on TEC permeability in the presence of conditioned media, which is likely due to the inhibitory effect observed upon using conditioned media only (Figure 2f). Direct endothelial leakiness is a general acute effect of both NMs, but it is not sufficient to predict metastasis. The final metastatic outcome depends on whether this permeability‐promoting effect is reinforced or counterbalanced by tumor‐cell‐ and additional TME conditions examined below.

### 3.3 NMs can Cause Changes in CAF Subtype Populations

**FIGURE 3:**
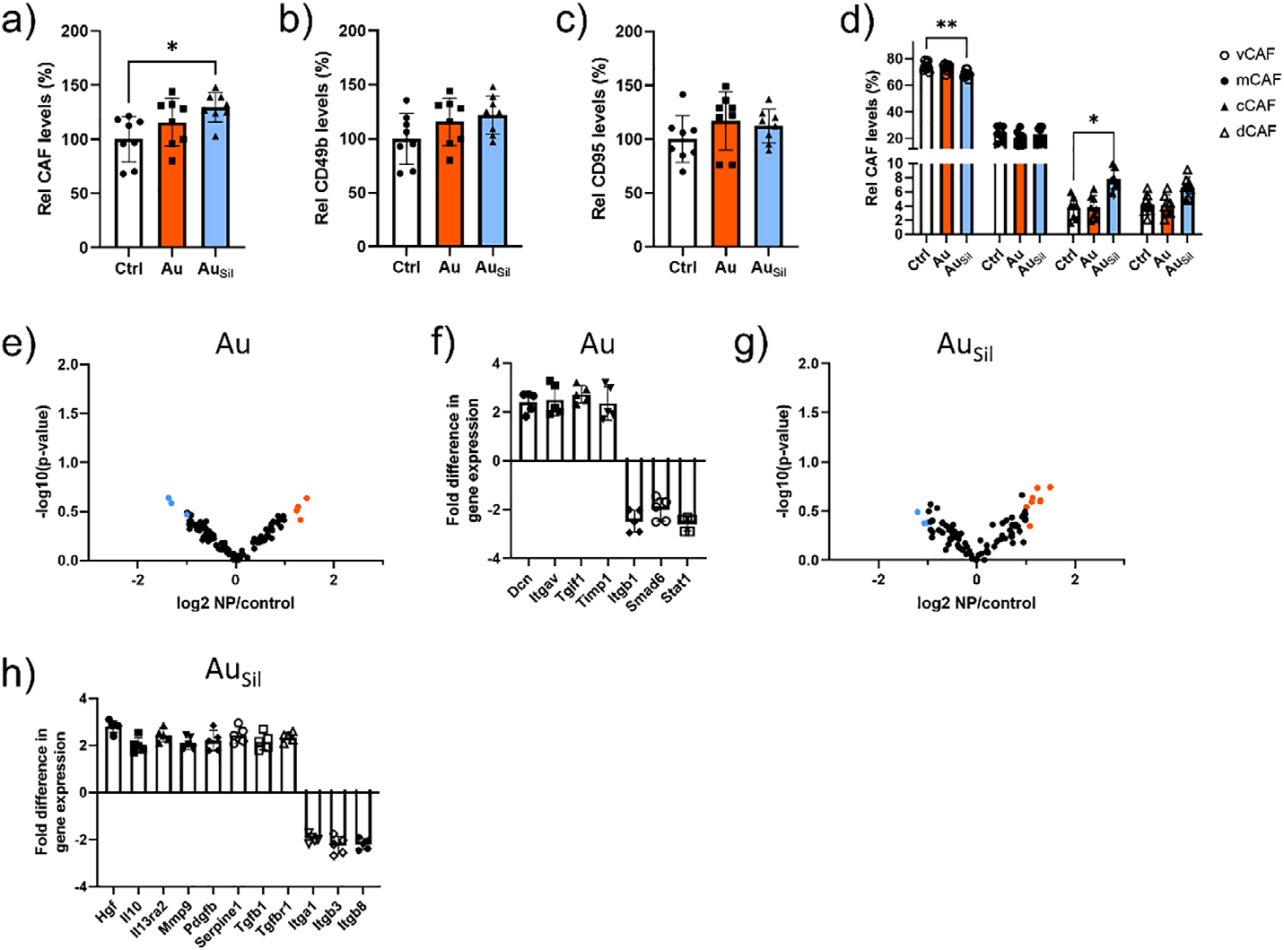
Au_Sil_ NMs affect total CAF levels and different CAF subtypes, but neither Au nor Au_Sil_ NMs result in direct CAF activation in 4T1 tumors. a) Histogram displaying the level of CD90.2^+^ CD45^−^ cells CAF cells obtained from tumors treated with the respective Au or Au_Sil_ NMs. b) Histogram representing the level of CD49b activation marker on CAFs obtained from tumors treated with the respective Au or Au_Sil_ NMs. c) Histogram representing the level of CD95 activation marker on CAFs obtained from tumors treated with the respective Au or Au_Sil_ NMs. d) Histogram displaying the relative levels of different CAF subtypes obtained from tumors treated with the respective Au or Au_Sil_ NMs. e) Volcano plot of genes involved in fibroblast signaling from CAF treated with Au NMs compared to CAF that were left untreated. Genes that were significantly up‐ or downregulated are indicated by orange or blue colour codes, respectively. f) Histogram representing the significantly up‐ or downregulated genes in CAF treated with Au NMs compared to CAF that were left untreated. g) Volcano plot of genes involved in fibroblast signaling from CAF treated with Au_Sil_ NMs compared to CAF that were left untreated. Genes that were significantly up‐ or downregulated are indicated by orange or blue colour codes, respectively. h) Histogram representing the significantly up‐ or downregulated genes in CAF treated with Au_Sil_ NMs compared to CAF that were left untreated. Animals in the control group were administered PBS only. Significant differences between a treated group and untreated controls are indicated where relevant (p < 0.05: *, p < 0.01: **; p < 0.001: ***; p < 0.0001: ****) based on ANOVA testing using GraphPad Prism 9 (n = 8, for (f) and (h) n = 5); data is represented as mean ± SD.

As the TEC results could not explain all observed effects, the influence of CAFs was studied. First, selection of CD45^−^ CD90.2^+^ CAFs revealed higher CAF levels upon Au_Sil_ NM exposure (Figure 3a). Both NMs had minor, but non‐significant increases on CAF activation levels, as indicated by CD49b and CD95 activation markers (Figure 3b,c) [41]. As CAFs are highly heterogeneous in function and origin [32], we looked into different functional subtypes of CAFs, based on a recent study on TNBCs [42]. Using this classification, CAFs are split into vCAFs (involved in angiogenesis and vascular remodeling), mCAFs (ECM matrix modulation), cCAFs (cell division), and dCAFs (cell differentiation and tissue development). Figure 3d shows that the CAFs are mainly composed of vCAFs and mCAFs with little amounts of cCAFs and dCAFs present. While Au NMs had no effect, Au_Sil_ NMs significantly reduced vCAFs and increased cCAF levels. It is unclear what the functional outcome of this would be, but it would hint toward vascular remodeling (vCAF) and increased CAF levels (cCAF). To study the functional outcome, gene expression studies toward fibroblast‐mediated signaling were performed, revealing several significantly up‐ and downregulated genes for both Au and Au_Sil_ NMs (Figure 3e–h). The NMs resulted in differently affected genes, where Au_Sil_ affected a broader set of genes. Au NMs mainly affected genes involved in TGFβ signaling (*Dcn, Tgif1, Smad6*), while Au_Sil_ NMs affected genes involved in inflammation (*Il10, Il13ra2*), cell adhesion (*Itga1, Itgb3, Itgb8*), and EMT (*Tgfb1*, *Serpine1*, *Mmp9*). When exposing TEC monolayers to conditioned media of NM‐treated CAFs, both NMs resulted in a trend toward increased TEC permeability, yet non‐significant (Extended Data Figure S5a). Tumor cells exposed to CAF‐conditioned media revealed no clear NM‐mediated effect on cell mobility (Extended Data Figure S5b). Overall, Au_Sil_ NMs altered CAF abundance, subtype distribution, and the expression of genes related to inflammation, adhesion, and EMT. However, CAF‐conditioned media did not induce a strong functional change in TEC permeability or tumor‐cell motility under the conditions tested. We therefore interpret CAF remodeling as a stromal response that may contribute to the Au_Sil_ ‐associated prometastatic TME, rather than as the dominant mechanism driving NM‐mediated metastasis.

### 3.4 TAM‐Rich and Hypoxic TME Conditions Amplify Au_Sil_‐Associated Metastatic Dissemination

**FIGURE 4:**
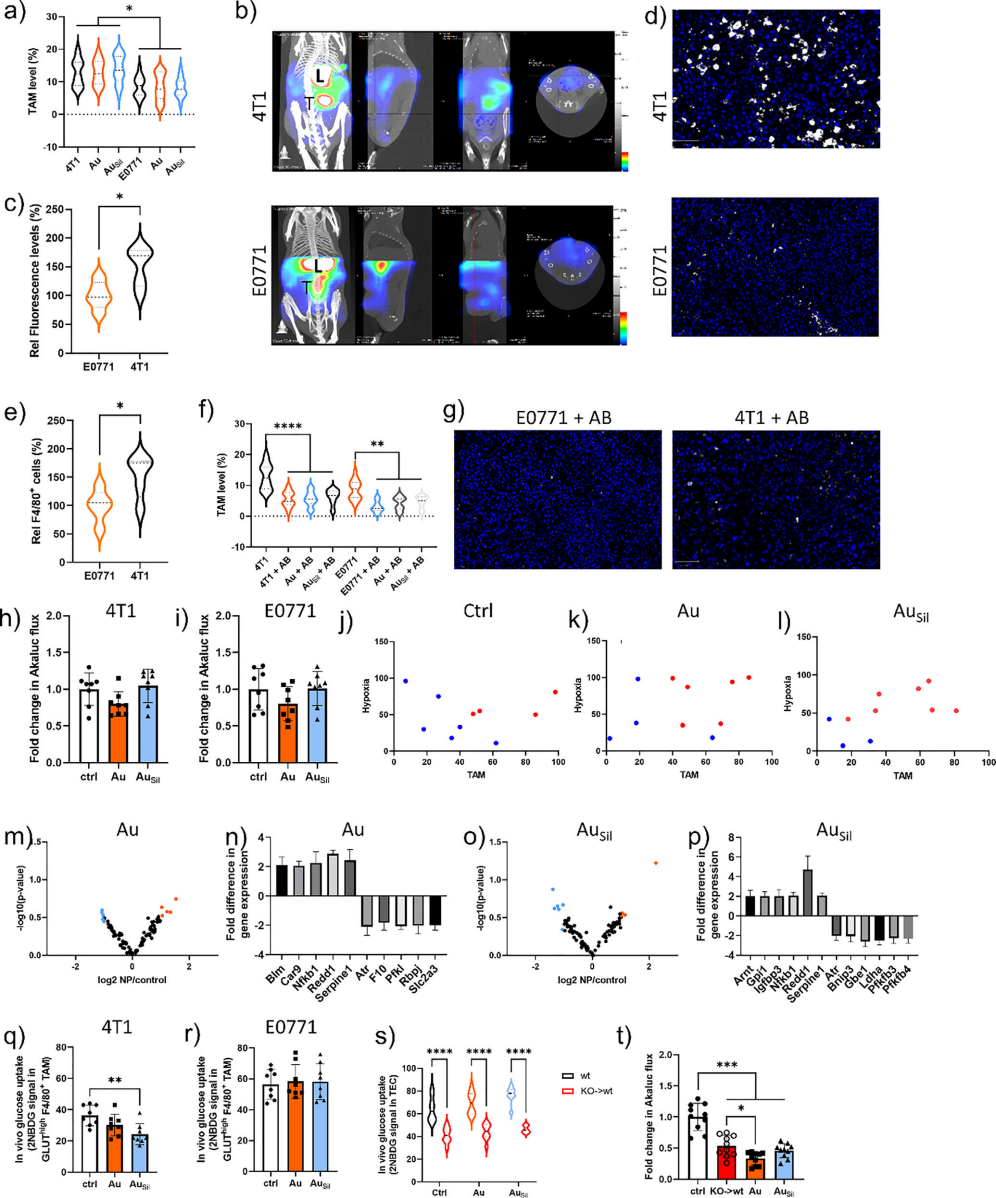
Hypoxic TAMs play a major role in NM‐mediated metastasis induction. a) Histogram displaying the level of TAMs relative to the total number of cells as determined by ImageStreamX Mark II analysis for 4T1 tumors (left 3 samples) and E0771 tumors (right 3 samples). b) Representative FLECT/CT images of mice having been grafted orthotopically with 4T1 (top) or E0771 (bottom) tumors. Images were acquired 25 days following tumor grafting after administration of fluorescently labeled nanoparticles with high uptake by phagocytic cells. The images show a 3D display, followed by a lateral and axial view. The L indicates the liver, T is to left of the tumor. c) Histogram displaying the quantitative fluorescence data obtained from FLECT/CT images (n = 3) acquired from animals bearing orthotopic 4T1 or E0771 tumors as described in b. The data are displayed relative to the level of E0771 TAMs (= 100%). d) Representative immunofluorescence images of tumor tissue sections obtained from mice having been grafted orthotopically with 4T1 (top) or E0771 (bottom) tumors. The sections were stained with anti‐F4/80 (white) and Hoechst nuclear counterstain. Scale bars: 50 µm. e) Histogram displaying the relative number of TAMs in 4T1 or E0771 tumors as determined by analysis of F4/80+ cells in immunofluorescent staining of tissues slices as described in d. f) Violin plots showing the relative level of TAMs expressed relative to the total number of cells as determined by ImageStreamX Mark II analysis for 4T1 tumors (left 4 samples) and E0771 tumors (right 4 samples) that had been treated with anti‐CSF1R antibody for 3 days prior to NM administration and isolation. g) Representative immunofluorescence images of tumor tissue sections obtained from mice having been grafted orthotopically with 4T1 (left) or E0771 (right) tumors and treated with anti‐CSF1R antibody for 3 days prior to tumor isolation. The sections were stained with anti‐F4/80 (white) and Hoechst nuclear counterstain. Scale bars: 50 µm. h,i) Akaluc luminescence signal measured over the lung area in tumor‐(h: 4T1; i: E0771) bearing mice that had been treated with anti‐CSF1R antibody for 3 days prior to NM administration. Tumors were then left to grow for another 7 days before surgical removal of the tumors at 25 days post‐grafting, and removal of the lungs 7 days following surgical tumor removal. j‐l) Dot plots expressing the level of TAMs and hypoxia and their influence on the level of metastasis in 4T1 tumor models for j) control, k) Au NM‐treated or l) AuSil NM‐treated tumors. The level of TAMs and hypoxia were linearly scaled from 1 (minimum) to 100 (maximum). Animals that were found to be positive for metastasis are indicated in red; those that are negative are indicated in blue. The cut‐off for occurrence of metastases was defined as the number EpCAM+ cells circulating in the blood exceeding 10/mL. m‐p) Gene expression data for isolated NM‐positive TAMs relative to untreated control TAMs for m,n) Au NMs and o,p) Au_Sil_ NMs. Volcano plots (m, o) are shown for 84 genes related to hypoxia‐signaling, where significantly expressed gens are indicated in orange (overexpression) or blue (underexpression). The significant genes are further indicated with their respective fold difference compared to untreated TAMs in n and p. q) Histogram indicating the in vivo glycolysis level of E0771‐derived TAMs determined by ImageStream analysis of isolated F4/80^+^ Glut1^high^ TAMs containing fluorescently tagged glucose analogue 2NBDG that had been administered intratumorally 60 mins prior to tumor isolation. Data are expressed as 2NBDG^+^ TAMs relative to the total TAM population. r) Histogram indicating the in vivo glycolysis level of TAMs determined by ImageStream analysis of isolated F4/80^+^ Glut1^high^ TAMs containing fluorescently tagged glucose analogue 2NBDG that had been administered intratumorally 60 mins prior to tumor isolation in E0771 tumors grafted in Redd1 KO BM‐transplanted animals. Data are expressed as 2NBDG^+^ TAMs relative to the total TAM population. s) Histogram indicating the in vivo glycolysis level of TECs determined by ImageStream analysis of isolated CD144^+^ TECs containing fluorescently tagged glucose analogue 2NBDG that had been administered intratumorally 60 mins prior to tumor isolation. Data are expressed as 2NBDG^+^ TECs relative to the total TEC population. t) Akaluc luminescence signal measured over the lung area in E0771‐bearing mice before isolation of the tumors at 25 days post grafting. The results are shown for E0771 tumors grafted in wild type Bl6 mice, or for E0771 tumors grafted in Bl6 having received Redd1 KO bone marrow grafts and then left either untreated (KO‐wt), or treated with Au or Au_Sil_ NMs. Animals in the control group were administered PBS only. Significant differences between a treated group and untreated controls are indicated where relevant (p < 0.05: *, p < 0.01: **; p < 0.001: ***; p < 0.0001: ****) based on ANOVA testing using GraphPad Prism 9 (n = 8; for (n) and (p) n = 3); data is represented as mean ± SD.

Regarding TAMs, neither of the NMs resulted in increased polarization, where most were found to lean toward the classical M_2_ phenotype (Extended Data Figure S5c–e). When supernatant of NM‐exposed TAMs was given to endothelial cells, permeability remained unaffected, and it also did not affect cancer cell migration (Extended Data Figure S5f–h). Interestingly, 4T1 tumors were found to have significantly higher TAM levels than E0771 tumors (Figure 4a). This was confirmed by preclinical imaging, where tumor‐bearing animals were given fluorescently labeled microspheres and quantitatively analyzed in view of their respective tumor uptake using 3D fluorescence emission computed tomography (FLECT). 4T1 tumors resulted in higher levels of fluorescent microspheres than E0771 tumors, and associated *ex vivo* histology of tumor tissues (Figure 4b–e). Treatment of the animals with anti‐CSF1R antibodies resulted in TAM depletion (Figure 4f,g, Extended Data Figure S5i), which in turn completely cancelled the effect of Au_Sil_ NMs on metastasis promotion (Figure 4h,i). Au NMs on the other hand were found to still reduce metastasis and CTC levels, however this effect was not always significant for every readout (Figure 4h,i; Extended Data Figure S6a–h). This was likely due to the fact that the overall metastasis level in TAM‐depleted tumors was lower than for normal wild‐type tumors, as M_2_‐TAMs are known to be involved in and promote metastasis [43].

More detailed analysis revealed that TAM levels alone could not explain the effects observed, but that apart from TAM levels, hypoxia also appeared to play a role in metastases, as long as high levels of TAMs are present (determined by in vivo optical imaging; Extended Data Figure S5i). Interestingly, the addition of Au and in particular Au_Sil_ NPs appear to promote metastases even in conditions where TAMs and hypoxia levels would be too low to be linked to metastases in control conditions (Figure 4j–l). These data suggest that even in conditions or relatively low TAM or hypoxia levels, the NMs, and in particular the AuSil NMs can promote metastases. Gene expression analysis of hypoxia‐related genes revealed significantly up‐ and downregulated genes for both Au and Au_Sil_ NMs (Figure 4m–p). The most pronounced gene of interest was *Redd1*, which has been described to promote tumor metastasis by uncontrolled blood vessel growth [44], and we found this was far more upregulated by Au_Sil_ NMs. In vivo glycolysis in TAMs was indeed found to be affected by Au_Sil_ NMs (Figure 4q). To understand the role of NM‐mediated REDD1 induction in hypoxic TAMs, we deleted Redd1 in macrophages by transplanting wild‐type (WT) recipient mice with REDD1 knockout (KO) bone marrow cells (KO→WT). Upon reconstitution, KO→WT chimeras displayed normal blood counts, comparable with those of WT→WT mice (Extended Data Figure S7a). We then orthotopically implanted E0771 cells and found that tumor growth itself was not significantly affected (Extended Data Figure S7b). As Redd1 is known to be involved in glycolysis [45, 46], which can have extensive effects on TAM physiology [44], the level of glycolysis in the TAMs was then studied. It was observed that glycolysis in TAMs was much higher and no longer affected by any NMs (Figure 4r). The elevated glycolysis in TAMs resulted in reduced glycolysis in TECs (Figure 4s) and this resulted in a significant reduction in metastasis compared to control mice (Figure 4t; Extended Data Figure S7c–i). Interestingly, Au_Sil_ NMs had no effect on metastasis levels in Redd1 KO models, while Au NMs further impeded metastasis levels and even increased tumor vessel length and maturity levels, which are known to impede tumor cell extravasation (Extended Data Figure S7c–i). These data suggest that TAM abundance and hypoxia determine whether the permeability‐promoting effects of NMs are converted into a measurable increase in metastasis. TAM depletion reduced the prometastatic effect of Au_Sil_ NMs, indicating that endothelial leakiness alone is not sufficient to drive the full metastatic phenotype in the absence of a permissive TAM‐rich microenvironment. Moreover, macrophage‐associated Redd1 loss abolished the metastasis‐promoting effect of Au_Sil_ NMs, supporting a functional role for the TAM–hypoxia–REDD1 axis as a dominant amplifier of NM‐associated metastasis. In contrast, Au NMs retained metastasis‐limiting effects in this setting.

### 3.5 Vessel Normalization and Hypoxia Inhibition Reduce Metastasis in NM‐Treated Tumors Under the Tested Conditions

**FIGURE 5:**
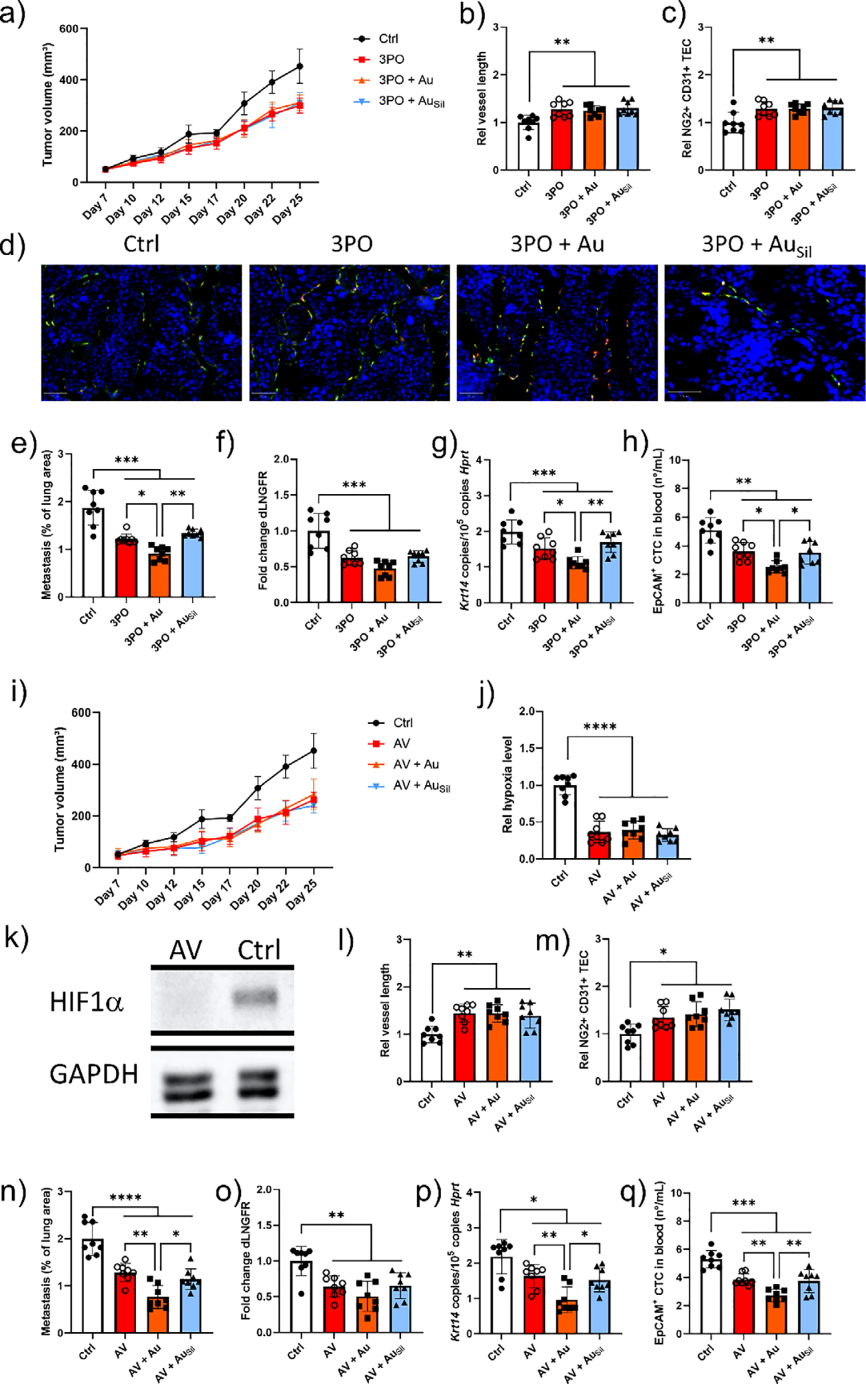
NM‐mediated metastasis can be inhibited by vessel normalization or reduced hypoxia signaling. a) Histograms expressing the tumor volume of untreated tumors or tumors in animals having been treated with 3PO to induce vessel normalization in the absence or presence of Au or Au_Sil_ NMs. b,c) Histograms displaying immunohistochemical analysis of tumor tissues analysed for CD31^+^ vessel length (b) or the level of NG2^+^ pericyte‐covered CD31^+^ vessels (c). Data are expressed relative to the vessel length of the pericyte coverage rate of untreated E0771 tumors. d) Representative images of tumor tissue sections obtained from control animals (left), or animals treated with 3PO (2^nd^ left), animals treated with 3PO and given Au NMs (2^nd^ right) or animals treated with 3PO and given Au_Sil_ NMs. The images display Hoechst (blue), CD31 (green) and NG2 (red); scale bars = 50 µm. e) The area taken up by metastatic nodules in the lung tissue slice as determined by H&E staining relative to the area of the entire tissue slice. f) Expression of the truncated dLNGFR receptor that was introduced into the tumor cells and determined by qPCR of lung tissue and expressed relative to the level of untreated tumor‐bearing mice. g) The level of Krt14 expression relative to that of housekeeping gene Hprt determined by qPCR of blood samples taken at 25 days post tumor grafting. h) The number of EpCAM^+^CD45^−^ cells detected in the blood at 25 days post tumor grafting expressed as the number of EpCAM^+^ cells per mL of sample and determined by ImageStream analysis. i) Histogram expressing the tumor volume of untreated tumors or tumors in animals having been treated with acriflavine (AV) to impede hypoxia‐mediated signaling in the absence or presence of Au or Au_Sil_ NMs. j) Histogram showing the relative in vivo hypoxia levels in tumors as determined by HypoxiSense imaging. The data are expressed for AV‐treated animals in the absence or presence of Au or Au_Sil_ NMs relative to that of untreated control animals. k) Representative Western Blot of tumor tissue obtained from untreated or? AV‐treated tumors and analyzed for expression of HIF1α complexes and GAPDH as housekeeping protein. l,m) Histogram displaying immunohistochemical analysis of tumor tissues analysed for CD31^+^ vessel length (l) or the level of NG2^+^ pericyte‐covered CD31^+^ vessels (m). Data are expressed relative to the vessel length of the pericyte coverage rate of untreated E0771 tumors. n) The area taken up by metastatic nodules in the lung tissue slice as determined by H&E staining relative to the area of the entire tissue slice. o) Expression of the truncated dLNGFR receptor that was introduced into the tumor cells and determined by qPCR of lung tissue and expressed relative to the level of untreated tumor‐bearing mice. p) The level of Krt14 expression relative to that of housekeeping gene Hprt determined by qPCR of blood samples taken at 25 days post tumor grafting. q) The number of EpCAM^+^CD45^−^ cells detected in the blood at 25 days post tumor grafting expressed as the number of EpCAM^+^ cells per mL of sample and determined by ImageStream analysis. Animals in the control group were administered PBS only. Significant differences between a treated group and untreated controls are indicated where relevant (p < 0.05: *, p < 0.01: **; p < 0.001: ***; p < 0.0001: ****) based on ANOVA testing using GraphPad Prism 9 (n = 8); data is represented as mean ± SD. In figures b,c,j,l,m, the data are expressed as normalized data relative to untreated control cells (= 1).

The data show a complex interaction between different TME components that can result in endothelial leakiness or tumor cell migration. As vessel leakiness is known to be a major cause of cancer cell extravasation, the influence of pharmacological tumor vessel normalization was studied, using 3‐(3‐pyridinyl)‐1‐(4‐pyridinyl)‐2‐propen‐1‐one (3PO) which has been shown to efficiently inhibit 6‐phosphofructo‐2‐kinase/fructose‐2,6‐biphosphatase 3 (PFKFB3) [47]. 3PO thereby reduces angiogenesis and promotes vessel maturity through impeding TEC glycolysis [48]. 3PO treatment slightly reduced tumor growth, but this was found not to be significant (Figure 5a). Vessel normalization has been successfully used to impede tumor metastasis [49], which was also true in our studies. 3PO was found to significantly increase tumor vessel length and vessel maturity while reducing lung metastases and tumor cell extravasation (Figure 5b–h).

Treatment of tumors with acriflavine, a potent inhibitor of hypoxia‐inducible factor 1α (HIF1α), also resulted in a minor decrease in tumor growth, followed by a potent drop in tumor‐associated hypoxic signaling (Figure 5i–k). Highly similar as for 3PO treatment, this resulted in significantly increased tumor vessel length and vessel maturity while reducing lung metastases and tumor cell extravasation (Figure 5l–q). Interestingly, in both treatment cases, Au NMs were found to further decrease metastasis levels up to significant levels for multiple readouts, while not having any influence on tumor vessel maturity or length itself. Together, these data indicate that vascular immaturity and hypoxia‐related signaling are conditions for NM‐associated metastasis promotion. Although direct NM–TEC interactions can increase endothelial permeability, this effect can be functionally reduced by stabilizing the vasculature or suppressing hypoxia signaling, suggesting that endothelial leakiness alone is not sufficient to determine the final metastatic outcome.

### 3.6 Au NMs can Impede Metastasis Through Reduced Tumor Cell Motility at Blood Vessels

**FIGURE 6:**
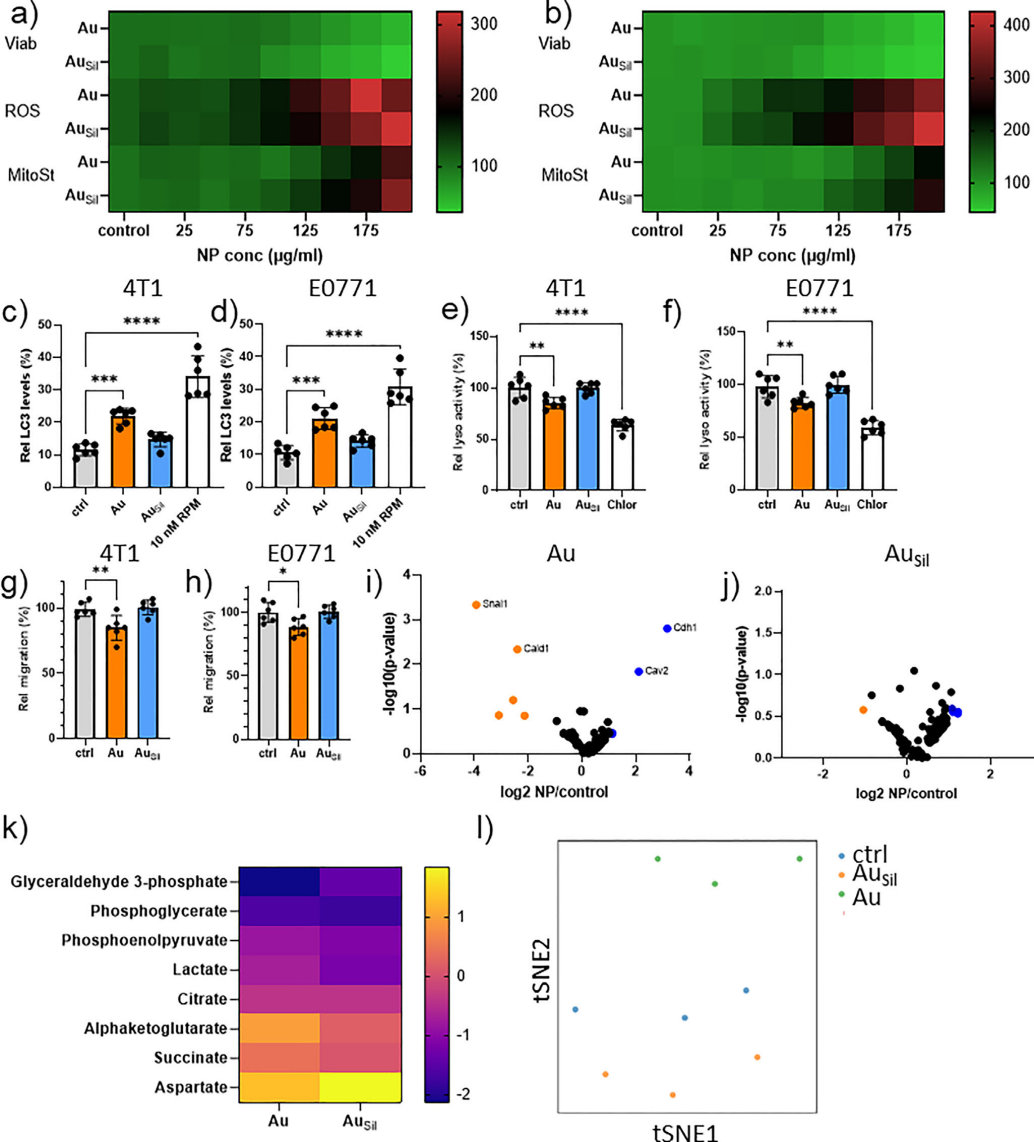
Au NMs affect cellular metabolism and lipid composition and impede tumor cell migration. a,b) Heatmaps of (a: 4T1; b: E0771) treated with different concentrations of Au or Au_Sil_ NMs and analyzed for cell viability, ROS, and mitochondrial health. c,d) Histogram displaying the number of (c: 4T1; d: E0771) cells displaying clear LC3 puncta, indicative of autophagosome formation. e,f) Histogram displaying the lysosomal activity of (e: 4T1; f: E0771) cells analyzed by measuring lysosomal phosphatase activity levels. Data are expressed relative to those of untreated control cells. g,h) Histogram displaying the migration capacity of (g: 4T1; h: E0771) cells through a Boyden chamber. i,j) Gene expression data for EMT and cellular mobility‐related genes represented as volcano plots of E0771 tumor cells treated with q) Au NMs or s) AuSil NMs and expressed relative to untreated tumor cells where significantly expressed genes are indicated in orange (overexpression) or blue (underexpression). k) Heatmap of metabolomics study on E0771 cells treated with Au or Au_Sil_ NMs and expressed relative to the values obtained for untreated control cells. The metabolites that were studied are involved in glycolysis and Krebs cycle and are expressed as the fold‐change relative to untreated control cells (= 0). l) A tSNE dimensionality reduction plot of E0771 cells left untreated or treated with Au or Au_Sil_ NMs and analyzed for lipidomics. A total of 3 samples were analyzed per condition. Control treatment groups received growth medium in the absence of NMs. Significant differences between a treated group and untreated controls are indicated where relevant (p < 0.05: *, p < 0.01: **; p < 0.001: ***; p < 0.0001: ****) based on ANOVA testing using GraphPad Prism 9 (n = 8).

Both 4T1 and E0771 cells were exposed to a wide concentration range of Au and Au_Sil_ NPs. Both cell types gave similar results, displaying a concentration‐dependent toxicity, increase in reactive oxygen species (ROS), and mitochondrial stress (Figure 6a,b). Using a ROS scavenger, viability and mitochondrial stress could be largely restored (Extended Data Figure S8a–f), suggesting that ROS was a major cause of the observed effects. Differential effects between the 2 NPs were observed when looking into autophagy and the cellular degradative capacity, where Au NPs (50 µg/mL) resulted in increased levels of autophagosomes (LC3) and lysosomal pH and decreased lysosomal degradation, which were not observed for Au_Sil_ NPs (Figure 6c–f; Extended Data Figure S8g–j). This is in line with previous reports, where Au NPs had been found to have an alkalyzing effect on lysosomal pH and hereby affect lysosomal enzyme activity [50]. Au NP, but not Au_Sil_, were also found to impact cell spreading, which affected focal adhesion formation and cellular migration capacity (Figure 6g,h; Extended Data Figure S8k–n), which is in line with previous studies that revealed Au NP interactions with cytoskeleton architecture and actin‐mediated signaling, including cell migration [51]. Gene expression studies revealed that both Au and Au_Sil_ NMs resulted in significant differences in EMT‐related genes (Figure 6i,j; Extended Data Figure S8o,p). While there were some similarities, such as overexpression of *Mitf1*, *Ptp4a1* and downregulation of *Twist1*, Au NMs affected more genes than Au_Sil_ NMs and seemed to mainly impeded EMT processes.

We then looked into metabolomics studies, where it was found that both Au and Au_Sil_ NMs had nearly identical effects on metabolites involved in glycolysis and Krebs cycle (Figure 6k), but Au NMs generally had a stronger effect. For fatty acids (FA), the overall trends between both NMs were again highly similar, where Au NMs generally had the strongest impact (Figure 6k). A more in‐depth lipidomics analysis revealed distinct differences in FA content in terms of carbon chain length and degree of saturation, which again, was far more pronounced for the Au than for the Au_Sil_ NMs (Figure 6l; Extended Data Figure S9). Together, these data revealed that at subcytotoxic conditions, both NMs, but in particular the Au NMs affected cellular functionality and wellbeing. Previous studies had indicated that Au NMs can regulate the synthesis of downstream metabolites through upstream genes in important metabolic pathways, and in doing so affect cell adhesion and cytoskeleton remodeling [52].

While there was an effect on tumor cell migration in vitro, the total number of tumor cells in vivo that effectively will interact with intravenously administered NMs directly was found to be far lower than 1% [14, 20]. As the majority of tumor cells will not have seen any Au NMs, we looked at how this low number of Au NM^+^ cells could significantly reduce cancer cell extravasation. To this end, fluorescently tagged Au NPs were used to target orthotopic E0771 tumor, after which the tumors were analyzed. Blood vessels in animals with Au NMs compared to control animals did not reveal any differences in vessel length or vessel maturity levels, nor were there any differences in the level of TAM, CAF, TEC, TIL or CC or in the level of ECM density. Interestingly, spatial analysis of NM distribution revealed that NM^+^ cells were in close proximity to the tumor blood vessels (Extended Data Figure S8q). As cell motility is known to be paramount to tumor cell dissemination [53], the reduced mobility of Au NM^+^ tumor cells close to the blood vessels may be sufficient to reduce metastases. Our data support a local counter‐regulatory mechanism in which the small fraction of Au NM‐positive tumor cells, enriched near blood vessels, displays reduced spreading and migration. Because tumor‐cell intravasation occurs at the vascular interface, impaired motility of this perivascular NM‐positive population may be sufficient to counterbalance the permeability‐promoting effect of Au NMs in selected tumor contexts. This mechanism appears weaker or absent for Au_Sil_ NMs, which may explain why Au_Sil_ ‐induced endothelial permeability more readily translates into increased metastasis.

Taken together, Au_Sil_ NMs increase endothelial permeability. In TAM‐rich and hypoxic tumors, this endothelial effect is amplified by TAM‐associated hypoxia/REDD1 signaling and reduced vessel maturity, allowing increased tumor‐cell intravasation and metastasis. Au NMs also induce endothelial permeability, but this is counterbalanced by tumor‐cell‐derived anti‐permeability signaling and reduced migration of perivascular Au‐positive tumor cells. Therefore, in less TAM/hypoxia‐permissive settings such as E0771, the counter‐regulatory effects of Au can dominate over the permeability trigger. CAF remodeling may contribute to a prometastatic stromal environment, especially after Au_Sil_ exposure, but the current functional data do not support CAFs as the dominant driver of NM‐induced metastasis. Although TAM abundance and hypoxia‐associated signaling explain a major part of the tumor‐model dependence observed here, other baseline differences between 4T1 and E0771 tumors, including intrinsic metastatic capacity, vascular organization and tumor‐cell‐intrinsic signaling, may also contribute and should be further dissected in future studies. In particular the composition of the TME composition seems to play a significant role, particularly regarding the level of TAMs as high levels of TAMs appear to promote tumor metastases, the extent of which seems to correlate with the level of oxidative stress elicited by the NMs. In a tumor model with low levels of TAMs, NMs may even reduce metastasis levels, but this was here only observed for the Au NMs. It remains unclear whether this is true for other NM types as well and what the underlying cause may be. One possible suggestion, based on our data here and other reports indicating that Au NMs have an intrinsic capability to reduce cell motility suggests less mobile tumor cells that may result in reduced intravasation of tumor cells. The contribution of other TME factors on reducing tumor metastases in low TAM tumors also remains to be further studied as this may hold interesting potential for a dedicated use of properly selected NMs in translational settings to minimize risks. We therefore suggest further studies are needed here, to further unravel the effect of our studied NMs on TME components or, for example, on extracellular vesicles, known to play a large role in intercellular communication and metastasis. From a translational perspective, these findings suggest that the safe design of inorganic nanomedicines should not rely solely on optimizing tumor accumulation or minimizing acute cytotoxicity. Instead, existing TME features, including tumor hypoxia, TAM abundance, endothelial permeability, and vessel maturity, should be considered as potential determinants of metastasis related risk. The inhibitory effects observed after tumor vessel normalization or hypoxia inhibition indicate that these pathways may serve as actionable checkpoints during nanomedicine development.

## Author Contributions

**Bella B. Manshian**: resources, supervision, Writing – review and editing, methodology, funding acquisition. **Tianjiao Chu**: methodology, investigation. **Mukaddes Izci**: investigation, methodology, writing – original draft, visualization. **Christy Maksoudian**: writing – original draft, investigation, methodology, visualization. **Xiaoqi Zheng**: writing – review and editing, validation, investigation, visualization. **Xueqi Fan**: investigation, writing – review and editing, validation, visualization. **Jonas Dehairs**: investigation, methodology, resources. **Irati Perez Gilabert**: investigation, methodology. **Stefaan J. Soenen**: conceptualization, funding acquisition, visualization, supervision, writing – review and editing, writing – original draft. **Filipa Goncalves**: methodology, investigation. **Kiana Buttiens**: writing – original draft, investigation, methodology, visualization. **Johan Swinnen**: resources, conceptualization, investigation, supervision.

## Conflicts of Interest

The authors declare no conflicts of interest.

## Supporting information




**Supporting File**: advs77310‐sup‐0001‐SuppMat.docx.

## Data Availability

All research data is provided in the main manuscript and accompanying supplementary information. Raw data files can be obtained from the author through the KU Leuven institutional data repository.
