# Supplementary material for: Inorganic Nanomaterials can Promote or Inhibit Tumor Metastasis Depending on Tumor Microenvironment Composition
Source: Adv Sci (Weinh). 2026 Aug 20:e77310. Online ahead of print. doi: 10.1002/advs.77310 (PMC13491526; doi:10.1002/advs.77310)
Supplement: Supplementary file 1 [file ADVS-9999-e77310-s001.docx]

**Supporting information**

**“Inorganic nanomaterials can promote or inhibit tumor metastasis depending on tumor microenvironment composition”**

Kiana Buttiens^1a^, Mukaddes Izci^1a^, Christy Maksoudian^1a^, Irati Perez Gilabert^1^, Tianjiao Chu^1^, Filipa Goncalves^1^, Jonas Dehairs^2,3^, Johan Swinnen^2,3^, Bella B. Manshian^2,4^, Stefaan J. Soenen^1,2,*^

1. NanoHealth and Optical Imaging Group, Department of Imaging and Pathology, KU Leuven, Belgium

2. Leuven Cancer Institute, KU Leuven, Belgium

3. Laboratory of Lipid Metabolism and Cancer, Department of Oncology, KU Leuven, Belgium

4. Translational Cell and Tissue Research Unit, Department of Imaging and Pathology, KU Leuven, Belgium

^a^These authors contributed equally

* s.soenen@kuleuven.be (Corresponding author)

***Supporting experimental section***

**Animals**

REDD1 KO mice were generated by Lexicon Corp (USA) exclusively for Quark Pharmaceuticals Inc and are the property of Quark Pharmaceuticals Inc. Balb/c and C57Bl6 mice were ordered from Charles River. For optical imaging studies in C57Bl6 mice, albino mice were used to enhance sensitivity.

**Bone marrow transplantation**

C57Bl6 mice of 5 weeks old were irradiated with 9.5 Gy using a Baltograph. Subsequently, 10^7^ bone marrow cells from REDD1 KO mice were injected intravenously via the tail vein. Tumor experiments were initiated 5 weeks after bone marrow reconstitution. To verify bone marrow efficacy, red and white blood cell count was determined using a haemocytometer on peripheral blood collected in heparin-coated capillary pipettes by retro-orbital bleeding.

**Tumor models**

The animal studies used orthotopic syngeneic tumor models in which Balb/c or C57Bl6 animals received 500,000 4T1 or E0771 cells, respectively, in saline as an injection into the 4^th^ mammary fat pad. All mouse surgical procedures and imaging were performed with the animals anesthetized by inhalation of 2% isoflurane. The condition of the animals was monitored every day and their weight was measured every other day. Tumors were measured with calipers every other day. When tumors reached the size of minimally 50 mm³ (approximately 7 days after tumor inoculation), the animals were divided into different groups of similar tumor size for further experiments and to receive NMs or other treatments. For euthanasia, animals were subjected to 5% isoflurane inhalation. To ensure death following isoflurane inhalation cervical dislocation was performed. Tumors were then removed from the animals and used for *ex vivo* analysis or for tissue dissociation to isolate cell populations of interest.

**Pharmacological treatment**

For tumor vessel normalization studies, animals were treated with a PFKFB3 inhibitor 3-(3-pyridinyl)-1-(4-pyridinyl)-2-propen-1-one) (3PO) (Sigma-Aldrich, Merck, Overijse, Belgium). The animals received intraperitoneal (i.p.) injections 3x a week (consecutive days) for a total of 14 days at 25 mg/kg 3PO in dimethyl sulfoxide (DMSO) (Sigma-Aldrich, Merck, Overijse, Belgium). The 3PO treatment started 4 days following NM administration (11 days post tumor cell grafting).

To investigate the effect of anti-hypoxia signaling, tumor-bearing mice received 2mg/kg acriflavine hydrochloride (MP Biomedicals) by i.p. administration on a daily basis for 14 consecutive days. The acriflavine treatment started 4 days following NM administration (11 days post tumor cell grafting).

Macrophage depletion was achieved by daily i.p. injection of 20mg/kg mouse anti-CSF1R antibodies (clone AFS98, BioXcell) or isotype IgG control (Sigma-Aldrich). Mice were pretreated for 3 days with anti-CSF1R before NM administration. Afterwards, mice continued to receive daily treatment for another 3 days and were then left to recover their macrophages until tumors could be removed. The efficiency of macrophage depletion was assessed by ImageStream analysis for F4/80 and immunohistochemistry staining against F4/80.

**Preclinical optical imaging**

For these studies, 4T1 tumors were grafted in Balb/c mice while E0771 tumors were grafted in albino C57Bl6 mice for enhanced sensitivity during imaging.

*Hypoxia*. Tumor hypoxia was measured by intravenous administration of Hypoxisense 680 (2 nmol/100 uL per mouse, Perkin Elmer, Life Sciences, Zaventem, Belgium) on day 24 following tumor grafting. The Hypoxisense 680 was measured *in vivo* 24h post-injection using the IVIS Spectrum (Perkin Elmer, Life Sciences, Zaventem, Belgium), after which the animals were sacrificed and tumors were collected for further processing.

*Longitudinal lung imaging*. The 4T1 or E0771 cells used were transduced with lentiviral vector encoding Akaluc for enhanced sensitivity. At 25 days following tumor cell grafting, the tumor was surgically removed and animals were carefully monitored for their wellbeing. At 7 days post primary tumor removal, the animals received Akalumine substrate (Sigma Aldrich, Belgium) by intravenous administration and were imaged using the IVIS Spectrum for the presence of any metastatic nodules. The lung were then removed for further ex vivo analysis as described below.

*In vivo determination of relative TAM levels*. Wild-type 4T1 or E0771 tumors were grafted orthotopically and at 24 days post tumor grafting, they received a single bolus administration of AngioSpark 680 fluorescent nanoparticles (Perkin Elmer, Life Sciences, Zaventem, Belgium). The animals were then left for 24h after which they were shaved completely and FLuorescence Emission Computed Tomography (FLECT) imaging combined with X-ray computed tomography (CT) was performed on the animals to visualize the lower abdomen and liver. FLECT/CT (InSyte, TriFoil Imaging, USA) enabled the quantitative detection of weaker signals in the orthotopic tumor in the presence of strong signal of the nearby liver. The photons could be quantified and the relative level of fluorescence in the tumor could be calculated by measuring the absolute photon count in the tumor region using VivoQuant software (Invicro, USA).

**Determination of tumor weight and metastatic nodules**

At 25 days post tumor cell grafting, tumors were removed and their weight was registered and samples were collected for histological examination. Lung metastatic nodules were contrasted after intratracheal injection of 15% India ink solution. Superficial metastatic nodules were assessed under a stereomicroscope.

**Tumor vascular perfusion and permeability**

When the tumor sizes reached to 80 mm^3^, the tumor vascular perfusion and permeability was determined by injecting intravenous (250 ug/ml) bovine serum albumin functionalized with Alexa Fluor 555 (BSA-AF555, Thermo Fisher Scientific, Ghent, Belgium) and measured *in vivo* using the IVIS Spectrum (Perkin Elmer, Life Sciences, Zaventem, Belgium) during 90 min with sequential measurements every 3 min (10 s, medium binning, excitation 500 nm and emission 580 nm). Measurements occurred immediately upon intravenous administration of BSA. Analysis was performed using the Living Image software (Perkin Elmer), where the kinetics of BSA fluorescence in the tumor was calculated and the same levels were calculated on the skull. The ratio of tumor over skull was then calculated resulting in a curve with a rapid initial slope, which then steeps off and turns into a gradual increase. Relative perfusion was defined as the maximal value obtained prior to the slope going down, and relative permeability was determined as the slope in the second, more gradual section of the curve.

**H&E staining of lung tissue for area of metastatic nodules**

Lung tissues were immediately frozen in OCT compound and 10 µm thick cryosections were cut and placed on histology slides. The tumor slides were air-dried without dehydration for 30’ at room temperature and washed with 1X Phosphate buffered saline (PBS, Gibco, Thermo Fisher Scientific, Ghent, Belgium) for 5’. The slides were then stained with hematoxylin (Sigma-Aldrich, Merck, Overijse, Belgium) for 3’, washed with deionized water obtained from a Milli-Q system (MQ; Millipore, France) water for 5’, incubated for 1’ in ethanol 80% (0.15% HCl), washed for 1’ with MQ and incubated for 30’’ with ammonium-containing water, followed by washing for 5’ with MQ water and 95% ethanol for 1’. The tumor slides were incubated for 1’ in eosin (Sigma-Aldrich, Merck, Overijse, Belgium) and next dehydrated in 95% ethanol for 5’, twice in 100% ethanol for 5’ each followed by washing in Xylene (Sigma-Aldrich, Merck, Overijse, Belgium) twice for 5’ each. Finally, the samples were mounted with DPX mounting medium (Merck, Overijse, Belgium). The area of metastatic tumor nodules in the lungs were then calculated using QuPath software.

**Analysis of truncated dLNGFR expression**

The 4T1 or E0771 cells used were transduced with lentiviral vector encoding dLNGFR, which is only expressed in the central nervous system. Collected lungs were snap-frozen in OCT compound and processed for qRT-PCR to determine the expression of dLNGFR.

**Analysis of circulating tumor cells**

At 25 days post cells grafting, blood samples were collected (minimum 0.5 mL) and centrifuged in heparin-containing tubes to separate plasma from serum (15 min at 3500 rpm). The serum was then depleted from CD45^+^ cells using magnetic separation beads (StemCell Technologies), and the remaining samples were split into 2 fractions, where one was evaluated for expression of epithelial marker *Krt14* (not present in blood cells), and household gene *Hprt* by qPCR. The other fraction was used to stain with anti-CD45 (FITC, Thermo Fisher) and anti-EpCAM (PE-Texas Red, Thermo Fisher) antibodies and analyzed using ImageStream analysis, where circulatory tumor cells were defined as EpCAM^+^CD45^-^ cells.

**Permeability of TEC monolayers**

Isolated TECs were seeded in 4 µm pore sized transwell inserts (2*10^4^ cell/insert) and grown in medium until the formation of a monolayer was formed. Media were then replaced with fresh media without phenol red, and FITC-conjugated dextran (40 kDa; Invitrogen, Belgium) was added to the medium in the transwell insert. Permeability was then measured by measuring FITC fluorescence level in the medium in the bottom well. Permeability was measured under a variety of conditions, including:

- TECs only in full media;
- TECs in the presence of Au or Au_Sil_ NMs (50 µg/ml) for 4h;
- TECs pretreated with 10 µM PP1 (Sigma Aldrich, USA) and exposed to Au or Au_Sil_ NMs (50 µg/ml) for 4h in the presence of PP1 (10 µM);
- TECs pretreated with 20 µM PitStop 2 (BioConnect, MedChemExpress) and exposed to Au or Au_Sil_ NMs (50 µg/ml) for 4h in the presence of PitStop 2 (20 µM);
- TECs cultured in Ibidi flow chambers and cultured under continuous flow in the presence of Au or Au_Sil_ NMs (50 µg/ml) for 4h. The TECs were exposed to a shear stress of 1 dyn/cm², corresponding to velocity of 1.33 ml/min in a unilamellar flow direction;
- TECs cultured for 4h in conditioned culture media obtained from 4T1 cells previously exposed to Au or Au_Sil_ NMs (50 µg/ml) for 24h. After exposure of 4T1 cells to the NMs, media was with fresh media without NMs and cells were left to grow overnight. Media were then collected and used to incubate the TECs for 4h.
- TECs cultured for 4h in conditioned culture media obtained from 4T1 cells previously exposed to Au or Au_Sil_ NMs (50 µg/ml) for 24h. After exposure of 4T1 cells to the NMs, media was with fresh media without NMs and cells were left to grow overnight. Media were then collected and used to incubate the TECs for 4h, to which Au or Au_Sil_ NMs (50 µg/ml) were added.

**Extracellular acidification rate of TECs**

Isolated TECs (150,000) were incubated overnight on Seahorse XF24 tissue culture plates (Seahorse Bioscience Europe). During the assay, the medium was replaced by unbuffered DMEM supplemented with 5 mM D-glucose and 2 mM L-glutamine, pH 7.4. The measurement of oxygen consumption and extracellular acidification was using the Seahorse XF24 analyser. Inhibitors required were used at the following concentrations: oligomycin (1 µM), FCCP (fluoro-carbonyl cyanide phenylhydrazone, 1.5 µM), antimycin A (1 µM) (all from Sigma-Aldrich).

**qPCR analysis of dedicated genes**

Tumor cells (4T1 or E0771) cells (1.5*10^6^) or isolated TAMs were exposed to Au or Au_Sil_ NMs (50 µg/ml) for 24h. RNA was extracted using the Qiagen RNeasy Mini Kit (Qiagen, Benelux BV, Netherlands) according to the manufacturer's instructions. Every RNA sample underwent DNase digestion to eliminate genomic DNA contamination using the RNase-Free DNase Set (Qiagen, Benelux BV, Netherlands). RNA samples were converted into first strand cDNA using the RT2 First Strand Kit (Qiagen Benelux BV, Netherlands) where Genomic DNA Elimination Mixture was applied according to the manufacturer's instructions and samples were PCR amplified. First strand cDNA was then used in the RT-PCR after samples were added to the RT2 qPCR Master Mix plus Sybr Green (Qiagen Benelux BV, Netherlands). The amplification of cDNA was performed in 10 μL reactions on a LightCycler 480 system (Roche Diagnostics). The mean cycle threshold (Ct) was determined by triplicate PCR runs, and the relative expression was normalized to that of the internal control (β-actin) via the 2−ΔΔCt method. Quantitative RT-PCR was performed using commercially available (Applied Biosystems and IDT) primers and/or probes for the studied genes (tumor cells: Ang1, Ang2, IFNγ, PDGF-D, SEMA3A, TGFβ1, TGFβ2, TSP-1, VEGF-A; TAMs: iNOS, IL1α, IL6, IL12, IL23, TNFα, Arg1, IL10, TGFβ). The sequence of primers and probes are available upon request.

**qPCR analysis of cellular pathways**

For gene expression studies in CAF, the following RT-PCR gene pathway array was used: the murine fibrosis signaling array (PAMM-120Z, Qiagen Benelux BV, Netherlands). For gene expression studies in tumor cells, the murine EMT signaling array was used (PAMM-090Z, Qiagen Benelux BV, Netherlands). Gene expression studies in hypoxic TAMs were performed using the hypoxia signaling array (PAMM-032Z). Isolated cells (1*10^5^) were either left untreated (controls) or incubated for 24 h with Au or Au_Sil_ NMs at 50 µg/ml for 24h. Next, cells were washed twice with PBS and harvested into centrifuge tubes. RNA was extracted using the Qiagen RNeasy Mini Kit (Qiagen, Benelux BV, Netherlands) according to the manufacturer's instructions. Every RNA sample underwent DNase digestion to eliminate genomic DNA contamination using the RNase-Free DNase Set (Qiagen, Benelux BV, Netherlands). RNA samples were converted into first strand cDNA using the RT2 First Strand Kit (Qiagen Benelux BV, Netherlands) where Genomic DNA Elimination Mixture was applied according to the manufacturer's instructions and samples were PCR amplified. First strand cDNA was then used in the RT-PCR after samples were added to the RT2 qPCR Master Mix plus Sybr Green (Qiagen Benelux BV, Netherlands). RT-PCR was performed on the iCycler iQ5 Thermal Cycler (Bio-Rad Laboratories N.V., Belgium). PCR array data was analysed using the ΔΔCt method via the GeneGlobe web portal. The PCR arrays contain 84 genes of interest, along with 5 household genes. Each RT^2^ Profiler PCR Array also includes control elements for: 1) Data normalization, 2) Genomic DNA contamination detection, 3) RNA sample quality and 4) General PCR performance. Analysis is only performed upon passing all 4 checks and where none of the household genes significantly differs from the other replicates or groups. For analysis, every control or condition is independently repeated three times.

**Analysis of cell secreted factors**

Tumor cells (4T1 or E0771) cells (1.5*10^6^) were exposed to Au or Au_Sil_ NMs (50 µg/ml) for 24h. Cells were centrifuged using a refrigerated centrifuge at 1000g for 20 min, and the supernatant was harvested. Commercial ELISA kits were then used against SEMA3A (CSB-EL020980MO, Cusabio, USA) and VEGFA (CSB-E04756m, Cusabio, USA) following the manufacturer’s protocols. In short, 100μl of diluted standard or sample is added per well, covered with the adhesive strip provided and incubated for 2 hours at 37°C. The liquid is removed and 100μl of Biotin-antibody (1:100 dilution) is added to each well. The wells are covered with a new adhesive strip and incubated for 1 hour at 37°C. Each well is gently aspirated and washed, which is repeated for a total of three washes. Wash by filling each well with the provided Wash Buffer (200μl). After the last wash, any remaining wash buffer is removed by aspirating. The plate is inverted and blotted against clean paper towels. 100μl of HRP-avidin (1:100 dilution) is added to each well. The microtiter plate is covered with a new adhesive strip and incubated for 1 hour at 37°C. The aspiration/wash process is repeated for five times as described above. 90μl of TMB Substrate provided with the kit is added to each well and incubated for 15-30 minutes at 37°C protected from light. 50μl of Stop Solution (provided with the kit) is added to each well, and OD values were then recorded at 450 nm using a microplate reader (SpectraMax M5).

**Analysis of CAF activation and subtypes**

Isolated CD90.2^+^ CD45^-^ CAF cells obtained from 4T1 tumors having previously been administered with Au or Au_Sil_ NM were further analyzed with expression against activation markers CD49b or CD95 using image-based flow cytometry (ImageStreamX Mark II, Amnis, Cytek, USA). Cells were stained with anti-CD49b rat monoclonal (PE-conjugated, ab25493, Abcam) or CD95 (PE-conjugated, REA453, Miltenyi Biotec) at a density of 1*10^5^ cells per sample in 50 µL volume of PBS pH 7.2, 2 mM EDTA, and 0.5% BSA (PEB buffer) at 2-8°C for 10min, followed by a washing step with PEB buffer.

For identification of CAF subtypes, isolated CAFs were stained with fluorescently tagged RNA probes (PrimeFlow RNA assay, Thermo Fisher Scientific, USA) against *Rgs5* (AF488), *Ccna2* (AF780), *Crabp1* (AF568) and *Trf* (AF647) according to manufacturer’s instructions. The RNA probes were then measured using FACS analysis (ImageStreamX MarkII, Amnis, Cytek, USA). The different subtypes were then roughly defined as follows: vCAF: *Rgs5*^+^ *Ccna2*^-^; mCAF: *Crabp1*^+^; cCAF: *Ccna2*^+^; dCAF: *Trf*^+^.

**Flow cytometry of TAM polarization**

Isolated TAMs from tumor-bearing animals having been injected with Au or Au_Sil_ NMs were centrifuged at 2500 rpm for 6 min after which the cells were stained with anti-CD163 antibody (SB436-anti-CD163 clone TNKUPJ, Thermo Fisher Scientific), anti-CD206 antibody (APC-anti-206 clone MR6F3 Thermo Fisher Scientific), anti-CD86 antibody (FITC-anti-CD86 clone GL1, Thermo Fisher Scientific) and anti-MHC II antibody (PE-Cy7-anti-MHC-II clone M5-114-15-2, Thermo Fisher Scientific) for 30 min at 4 °C. The cells were then centrifuged again, and resuspended in PBS after which they were run on the ImageStream X Mark II (Merck, Belgium) for analysis. Using iDEAS software, focused and single cells were selected and analysed for expression levels of the different markers. As controls, TAMs were isolated from tumor-bearing animals not having received any NMs (negative control) or were seeded at 1*10^5^ cells and stimulated for 24 hrs with 25 ng ml^–1^ IFNγ and 10 ng ml^–1^ LPS (M1 control) or 40 ng ml^–1^ IL-4 and 20 ng ml^–1^ IL-13 (M2 control).

**Tumor cell migration**

The 4T1 and E0771 cells were seeded in 25 cm² tissue culture flasks at 1 * 10^5^ cells/flask and allowed to settle overnight. Next, cells were incubated with fresh media (10 ml) containing the Au or Au_Sil_ NMs at 50 μg/ml for 24 h. Media were removed, cells washed twice with ice-cold PBS, after which they were reseeded in new well plates containing 8 μm-pore Boyden chambers (Cell Biolabs Inc, San Diego, CA, USA). In the upper compartment of the Boyden chamber, the cells were given in serum-free media, while the lower compartment contained full media with 10% FBS. Cell migration was then determined in accordance with the manufacturer’s instructions by counting the cells that during 4h had reached the lower compartment.

**High‑content analysis of cell‑nanoparticle interaction studies**

Both 4T1 or E0771 cells were seeded at 7500 cells/well in a 96 well plate (Nunc, Belgium). Cells were allowed to attach overnight in a humidified atmosphere at 37 °C and 5% CO_2_, after which the cells were incubated with either the Au or Au_Sil_ NPs for 24 h in full growth medium. For cellular exposure studies, cells were incubated with the NPs at 10, 25, 50, 75, 100, 125, 150, 175 and 200 μg/ml. Each experiment was performed in three independent repeats and data were analyzed using full data sets of the different repeats. The cells were analyzed for viability, reactive oxygen species (ROS), mitochondrial stress, LC3 puncta formation, lysosomal pH, cell area and FAC area using an InCell Analyzer 2000 as described below, in line with established protocols as detailed elsewhere.^1-3^

*Cell viability and membrane damage*

Following cellular exposure to the NPs, the cells were washed twice with phosphate buffered saline (PBS; Gibco, Invitrogen, Belgium) and treated with 2 µM fixable Live-Dead Green dead cell stain (Molecular Probes, Life Technologies Europe, BV, Belgium) in 250 µl/well of PBS (with Ca2+ and Mg2+) and incubated in the dark for 30 min at room temperature. Next, the staining media was aspirated, cells were washedgently with PBS (3x) fixed with 4% paraformaldehyde (PFA) for 15 min at room temperature. The fixative was aspirated and cells were washed three times with PBS. Cells were then counterstained using Hoechst 33342 Nuclear stain (20 µg/ml PBS in 250 µl/well) for 15 min at ambient temperature in the dark. The nuclear counterstain was then removed, cells were washed three times with PBS and 500 µl of PBS was added to every well, after which the plates were analyzed using the InCell 2000 analyzer (GE Healthcare Life Sciences, Belgium). During acquisition, a minimum of 50 fields per well were imaged using a 20x objective for the following channels: UV/blue for Hoechst nuclear stain, FITC/FITC for the Live-Dead Green dead cell stain. Data analysis was then performed on the InCell Investigator software (GE Healthcare Life Sciences, Belgium) using in-house developed protocols, using a minimum of 5000 cells/condition. The level of cell viability was calculated as follows: First, cells were segmented based on the Hoechst stain and the perinuclear region was determined by enlarging the nuclear stain 2.5-fold and using the original Hoechst stain images as seed images. For cell viability based on cellular exposure concentrations, cell viability was calculated by determining the number of total cells minus the number of dead cells (dead cells are defined as cells with clear green nuclei, where the intensity is minimally 3-fold above noise level and having an area of minimally 2 µm²). These values were then normalized to control values (100%) to indicate the degree of cell viability.

For membrane damage, the analysis occurred similarly. All green dots in the perinuclear area

with a minimum intensity of 3-fold above the noise level and with a size of minimally 0.1 µm² but smaller than 2 µm² were selected. The ratio of this value with the value obtained for control cells was then given to indicate the level of membrane damage.

*Oxidative stress*

After labeling the cells, they were washed (3x) with 500 µl PBS/well, after which the cells were incubated with 500 µl full media containing 5 µM CellROX Green (Molecular Probes, Invitrogen, Belgium) for 30 min at 37°C. The staining solution was aspirated, cells were

washed with 500 µl PBS/well (3x) and fixed with 4% PFA for 15 min at room temperature. The fixative was removed, cells were washed (3x) with PBS (500 µl/well) after which 500 µl of PBS was added per well and plates were kept at 4°C in a dark container until they were needed for analysis using the InCell 2000 high-content imaging system. For acquisition, the following channels were selected: UV/blue for Hoechst nuclear stain, FITC/FITC for the CellROX Green stain. Data analysis was then performed on the InCell Investigator software (GE Healthcare Life Sciences, Belgium) using in-house developed protocols, using a minimum of 5000 cells/condition. The level of oxidative stress was then calculated as follows: First, cell nuclei were segmented based on the blue channel (Hoechst). As CellROX Green localizes in the nucleus upon oxidation, the intensity of light emitted in the green channel was then determined for every area of the corresponding nuclei. The intensity of every nucleus was then calculated for the green channel and normalized to the intensity level of untreated control cells (100%).

*Mitochondrial stress*

After labeling the cells, they were washed (3x) with 500 µl PBS/well, after which the cells were stained in 250 µl PBS (containing Ca2+ and Mg2+) containing 200 nM MitoTracker Red CMXRos (Molecular Probes, Invitrogen, Belgium) and incubated for 30 min at room temperature. The staining solution was then aspirated, cells were washed with 500 µl PBS/well (3x) and fixed with 4% PFA for 15 min at room temperature. The fixative was removed, cells were washed (3x) with PBS (500 µl/well) after which 500 µl of PBS was added per well and plates were kept at 4°C in a dark container until they were needed for analysis using the InCell 2000 high-content imaging system. For acquisition, the following channels were selected: UV/blue for Hoechst nuclear stain, DsRed/DsRed for the MitoTracker Red CMXRos stain. Data analysis was then performed using the InCell Investigator software (GE Healthcare Life Sciences, Belgium) using in-house developed protocols, analyzing a minimum of 5000 cells/condition. The mitochondrial stress was then calculated as follows: First, cell nuclei were

segmented based on the blue channel (Hoechst). Then, the DsRed/DsRed channel was segmented, using the nuclear target channel as seed images. Based on the segmented mitochondrial images, the overall area of cellular mitochondria were calculated, for any dot in

the mitochondrial channel that had an intensity of minimum 3-fold higher than the background

noise level. The total area of cellular mitochondria was determined as a marker for mitochondrial stress, where damaged mitochondria change shape, turning from an elongated to a more spherical morphology. The total area of cellular mitochondria was then normalized to the area of mitochondria in untreated control cells (100%). For mitochondrial ROS, the level of fluorescence intensity of the segmented mitochondria was determined. The intensity of the mitochondrial signal was then normalized to the intensity level of untreated control cells (100%).

*Cell morphology, skewness and focal adhesions*

After cellular exposure to the NPs, cells were washed (3x) with 500 µl PBS/well and fixed for 15 min at room temperature with 4% PFA. The fixative was then aspirated, cells were washed (3x) with PBS (500 µl/well) after which cells were permeabilised with 250 µl/well of Triton X-100 (1%) for 10 min at room temperature. Cells were then blocked with 10% serum containing PBS for 30 min at room temperature. Next, cells were stained using 200 µl of staining solution per well (15 µl of 1/100 dilution of the stock in blocking buffer) of ActiStain 555 (Tebu-Bio, Belgium) and anti-vinculin antibody (1:200, Abcam, Cambridge, UK, #ab18058) and incubated for 90min in the dark at room temperature, followed by 1 h incubation at ambient temperature with secondary AlexaFluor 488-conjugated goat anti-mouse antibody (1:250, Molecular Probes, #H32720). The staining solution was aspirated, cells were washed (3x) with PBS (500 µl/well) after which 500 µl fresh PBS was added to each well and the plates were kept at 4°C in a dark container until analyzed using the InCell 2000 high-content imaging system. For acquisition, the following channels were selected: UV/blue for Hoechst nuclear stain, FITC/FITC for the vinculin stain and DsRed/DsRed for the actin stain. Data analysis was then performed on the InCell Investigator software (GE Healthcare Life Sciences, Belgium) using in-house developed protocols, using a minimum of 5000 cells/condition. The size of the cells was calculated as follows: First, cell nuclei were segmented based on the blue channel. Cells were then segmented using the DsRed channel, where any holes in the cells were filled up and included. Cells on the border of the field of view were excluded from the analysis. The segmentation was based on the blue channel as seed channel for the nucleus. The total area of every individual cell was then determined. For determination of skewness (*i.e*. the shape of the cells, being the ratio of cell width over cell length), the same approach was used. After segmentation, the “form factor” was calculated which provides the ratio of the cell width over

cell length. This value will always be between 0 (straight line) and 1 (perfect circle). For both parameters, the values obtained were then normalized to the values obtained for untreated control cells (100%). Analysis of focal adhesions occurred through the vinculin stain in the FITC/FITC channel, where any green spots having an intensity of minimum three times that of the noise level and an area of minimally 0.2 µm² (to avoid detection of cytoplasmic vinculin) and that were localized within the area (based on actin segmentation), were considered. Then, the total area of vinculin positive spots per cell were calculated and expressed relative to the total cellular area (100%).

*Autophagy*

After cellular exposure to the NPs, cells were washed (3x) with 500 µl PBS/well and fixed for 15 min at room temperature with 4% PFA. The fixative was then aspirated, cells were

washed (3x) with PBS (500 µl/well) after which cells were permeabilised with 250 µl/well of Triton X-100 (1%) for 10 min at room temperature. Cells were then blocked with 10% serum containing PBS (blocking buffer) for 30 min at room temperature. Next, cells were stained using 200 µl of staining solution per well consisting out of primary mouse anti-LC3 antibody (1/400 dilution in blocking buffer; Cell Signalling Technologies, Belgium) and incubated for 90 min in the dark at room temperature. The primary antibody solution was aspirated, cells were washed (3x) with blocking buffer (500 µl/well) after which 200 µl of secondary AF488-

conjugated goat anti-mouse IgG antibody (1/250 dilution in blocking buffer; Molecular Probes, Belgium) was added to each well and plates were incubated in the dark for 60 min at room temperature. Following this, the incubation media was aspirated, cells were washed (3x) with PBS (500 µl/well), after which 500 µl fresh PBS was added to each well and the plates were kept at 4°C in a dark container until analyzed using the InCell 2000 high-content imaging system. For acquisition, the following channels were selected: UV/blue for Hoechst nuclear stain, FITC/FITC for the LC3 stain. Data analysis was then performed on the InCell Investigator software (GE Healthcare Life Sciences, Belgium) using in-house developed protocols, using a minimum of 5000 cells/condition. The level of autophagy was calculated as follows: First, cell nuclei were segmented based on the blue channel. Using the FITC channel, the cell cytoplasm was then selected, and cells were segmented, where any holes in the cells were filled up and included and any cells on the border of the field of view were excluded from the analysis. The segmentation was based on the blue channel as seed channel for the nucleus. Using the original FITC/FITC channel, any green dots having an intensity of minimum twice that of the noise level and that were localized within the cytoplasm were segmented, where multiple green dots could be localized within a single cell cytoplasm. Then, the cellular intensity of the green channel was measured for every cell, after which this value was normalized to the control value (100%).

**Evaluation of lysosomal activity**

The TNBC cell lines 4T1 and E0771 were seeded in 25 cm² tissue culture flasks at 1 * 10^5^ cells/flask and allowed to settle overnight. Next, cells were incubated with fresh media (10 ml) containing the Au or Au_Sil_ NMs at 50 μg/ml for 24 h. Media were removed, cells washed twice with ice-cold PBS, after which cell lysates were prepared and acid phosphatase activity was measured according to the manufacturer’s instructions (Acid Phosphatase assay kit, Sigma-Aldrich, St. Louis-MO, USA). The results obtained for the NM-treated samples were normalized against the value obtained for untreated control cells at identical protein levels.

**In vivo glycolysis**

To characterize glucose uptake by TAMs or TECs *in vivo*, orthotopic 4T1 tumor-bearing mice were injected intratumorally with 10 μg of 2-NBDG (2-(N-(7-Nitrobenz-2-oxa-1,3-diazol-4-yl)Amino)-2-Deoxyglucose, Invitrogen) in 250 μl spread over 5 different injections. The mice were sacrificed after 30 min and the tumors were dissected and dissociated in a single cell suspension. Green fluorescent 2-NBDG signal intensity was measured by FACS in F4/80^+^ cells (TAMs) and CD31^+^ cells (TECs) and normalized for the tumor weight.

**Tumor analysis via ImageStream and histology**

The delivery of Au and Au_Sil_ NMs was evaluated using a multiparametric approach, in which NM delivery was measured using inductively-coupled plasma mass spectrometry (ICP-MS), while cellular NM distribution was visualized using darkfield flow cytometry (ImageStreamX MarkII). Tumor size, ECM density, level of TAMs, CAFs, TECs, TILs and cancer cells were evaluated by image-based flow cytometry and histology. A detailed description of all assays and markers are described below based on established protocols detailed in previous work.^4,5^

*Histology, immunostainings and morphometric analysis*

Mouse tissues were dehydrated after being fixed for minimum 48 h in 4% PFA at 4 °C. The tissues were after subsequently embedded in OCT compound (Sakura-Finetek, CA, USA) and frozen at -80 °C. Tissue slices of 10 µm were cut using the Cryostar NX70 (Thermo Fisher Scientific, Ghent, Belgium) and placed on glass microscope slides (VWR, PA, USA). For morphometric analyses, optical fields (40 × magnification) of the whole sections were taken by the high content screening microscope Nikon-Marzhauser Slide Express (Märzhäuser Wetzlar GmbH & Co. KG, Wetzlar, Germany) or Vectra Polaris multispectral imaging system (Perkin Elmer, Life Sciences, Zaventem, Belgium) and analysed using QuPath. The images were automatically stitched together to cover the entire slide and were saved as.OME TIFF of.qptiff file format for further analysis.

*Hematoxylin and eosin staining for determination of tumor necrosis and tissue damage*

Hematoxylin and eosin (H&E) staining was performed on 10 µm thick OCT-embedded frozen tissues obtained as described above. The tumor slides where first air-dried without dehydration for 30’ at RT and washed with 1X Phosphate buffered saline (PBS, Gibco, Thermo Fisher Scientific, Ghent, Belgium) for 5’. The slides were then stained protected from light with hematoxylin (Sigma–Aldrich, Merck, Overijse, Belgium) for 3’, washed with deionized water obtained from a Milli-Q system (MQ; Millipore, France) water for 5’, incubated for 1’ in ethanol 80% (0.15% HCl), washed for 1’ with MQ and incubated for 30’’ with ammonium-containing water, followed by washing for 5’ with MQ water and 95% ethanol for 1’. The tumor slides were incubated protected from light for 1’ in eosin (Sigma–Aldrich, Merck, Overijse, Belgium) and dehydrated after in 95% ethanol for 5’, twice in 100% ethanol for 5’ each followed by washing in Xylene (Sigma–Aldrich, Merck, Overijse, Belgium) twice for 5’ each. Finally, the samples were mounted with DPX mounting medium (Merck, Overijse, Belgium).

Tumor necrosis was then expressed as the percentage of the total tumor area as determined on H&E-stained sections. For this, the images were loaded as Brightfield (H&E) images, and a thresholder was created to classify pixels for eosin (tumor tissue) or background signal. This was followed by creating a thresholder for classifying pixels for hematoxylin signal, to discriminate healthy from necrotic tissue. The tumor area was then calculated based on the eosin stain, while the area of the healthy tissue is then calculated based on the hematoxylin stain. The relative level of necrosis is then calculated as follows: ((eosin area – hematoxylin area)/eosin area)*100 and expressed as percentage for all tissue sections and all tumors.

*Picrosirius red staining for determination of tumor extracellular matrix level*

The tumor slides were air-dried without dehydration for 30’ at RT and washed with 1X PBS (Gibco, Thermo Fisher Scientific, Ghent, Belgium) for 5’. The tumor samples were stained with Picrosirius red (PSR, Abcam, Cambridge, UK) and Fastgreen (0.1 g FCF in acidified PSR solution, Sigma-Aldrich, Merck, Overijse, Belgium) for 1 h at 4 °C and rinsed twice with 0.5% acidified water followed by washing in Ethanol absolute for 30″ one time and for 5’ two times. The samples were cleaned twice in Xylene for 5’ and mounted with DPX mounting medium (Merck, Overijse, Belgium).

Tumor extracellular matrix content was expressed as the percentage of the total tumor area. For analysis in QuPath, the images were loaded as Brightfield (H-DAB) images, and the images were preprocessed and stain vectors were estimated and adjusted to represent the PSR and Fastgreen signal, respectively. Using the combined colours, a thresholder was created to classify pixels for tumor tissue or background signal. This was followed by creating a thresholder for classifying pixels for PSR signal, to discriminate collagen fibres from other tissue. The tumor area was then calculated based on the combined stain, while the area of the collagen is then calculated based on the PSR stain. The relative level of necrosis is then calculated as follows: (PSR area/total area)*100 and expressed as percentage for all tissue sections and all tumors.

*Immunohistochemistry*

The tumor samples were air-dried at RT for 30’ without dehydration and were washed in 1X PBS for 5’. The samples were fixed in 100% cold MeOH for 6’ at -20 °C, washed for 5’ in 1XPBS, and incubated for 15’ with Proteinase K (1:500, in 1XPBS, Promega B.V., Leiden, The Netherlands) at 37 °C. After washing the slides 5’ with 1XPBS, the slides were blocked for 1 h at RT with 1XPBS + 10% normal goat serum (NGS, 60.0 mg/mL, ThermoFischer Scientific, Ghent, Belgium) and 1%FBS and washed again twice with 1XPBS for 5’ each. The samples were blocked with Avidin (0.001%, Avidin from egg white, Sigma-Aldrich, Merck Chemicals, Overijse, Belgium) for 20’, followed by washing with 1XPBS two times for 2’ each and blocked again with Biotin (0.001%, Sigma-Aldrich, Merck, Overijse, Belgium) for 20’ and washed twice for 2’ in 1XPBS each. The samples were incubated with anti-CD31 antibody (1:25, in 1XPBS + 1%NGS, Abcam, Cambidge, UK) overnight at 4 °C.

After leaving the samples for 20’ at RT, the samples were washed twice in 1XPBS for 5’ each, blocked for 20’ with hydrogen peroxidase (3%, Alexa Fluor 594 Tyramide SuperBoost Kit, Invitrogen, Thermo Fisher Scientific, Ghent, Belgium), washed twice in 1XPBS for 5’ each and incubated with Goat anti-Rat-Biotin (1:300, in 1XPBS + 1%NGS, Jackson ImmunoResearch Europe Ltd, Ely, UK) at RT for 1 h.

The samples were washed twice in 1XPBS for 5’ each, incubated with streptavidin-HRP (1:150, in 1XPBS + 1%NGS, Invitrogen, ThermoFischer Scientific, Ghent, Belgium) for 30’ at RT, washed again two times in 1XPBS 5’ each and incubated with Alexa Fluor Tyramide 594 (1:100, Alexa Fluor 594 Tyramide SuperBoost Kit, Invitrogen, ThermoFischer Scientific, Ghent, Belgium) + hydrogen peroxidase 3% (1:100, Alexa Fluor 594 Tyramide SuperBoost Kit, Invitrogen, Thermo Fisher Scientific, Ghent, Belgium) + Tris Buffer HCl pH 7,4 (1:1) for 10’ at RT. The reaction was stopped using Stop Reagent (1:11, in 1XPBS, Alexa Fluor 594 Tyramide SuperBoost Kit, Invitrogen, ThermoFischer Scientific, Ghent, Belgium) for 2’ at RT, washed three times in 1XPBS for 5’ and incubated with anti-laminin (1:200, in 1XPBS + 1%NGS, Sigma- Aldrich, Merck Chemicals, Overijse, Belgium) or with anti-neural/glial antigen 2 (NG-2, 1:100, in 1XPBS + 1%NGS, Abcam, Cambidge, UK), overnight at 4 °C. The samples are from here on divided in two parts; samples stained with the primary anti-CD31 antibody together with the primary anti-laminin antibody, and samples stained with the primary anti-CD31 antibody together with the primary anti-NG2 antibody.

*Anti-CD31 and anti-laminin co-staining*

The samples were washed twice in 1XPBS for 5’ each, incubated with Goat Anti-Rabbit-AF488 (1:1000, in 1XPBS + 1%NGS, Invitrogen, Thermo Fisher Scientific, Ghent, Belgium) for 1 h at RT, washed twice in 1XPBS for 5’ each and incubated for 10’ at RT with Hoechst (1:1000, in 1XPBS, Invitrogen, Thermo Fisher Scientific, Ghent, Belgium). Finally, the samples were washed twice in 1XPBS for 5’ each, mounted with Fluoromont (Sigma-Aldrich, Merck Chemicals, Overijse, Belgium), air-dried for 30’ with dehydration and sealed the cover slides with transparent nail polish.

*Anti-CD31 and anti-NG2 co-staining*

The samples were washed twice in 1XPBS for 5’ each, incubated with Goat Anti-Rabbit IgG secondary antibody poly HRP (1:1, Alexa Fluor 488 Tyramide SuperBoost Kit, goat anti-rabbit IgG, Invitrogen, Thermo Fisher Scientific, Ghent, Belgium) 1 h at RT, washed twice in 1XPBS for 5’ each, incubated with Alexa Fluor Tyramide 488 (1:100, Alexa Fluor 488 Tyramide SuperBoost Kit, goat anti-rabbit IgG, Invitrogen, Thermo Fisher Scientific, Ghent, Belgium) + hydrogen peroxidase 3% (1:100, Alexa Fluor 488 Tyramide SuperBoost Kit, goat anti-rabbit IgG, Invitrogen, Thermo Fisher Scientific, Ghent, Belgium) + Tris Buffer HCl pH 7,4 (1:1) for 10’ at RT, incubated after for 2’ at RT with Stop Reagent (1:11, in 1XPBS, Alexa Fluor 488 Tyramide SuperBoost Kit, goat anti-rabbit IgG, Invitrogen, Thermo Fisher Scientific, Ghent, Belgium), washed three times in 1XPBS for 5’ each and incubated for 10’ at RT with Hoechst (1:1000, in 1XPBS, Invitrogen, Thermo Fisher Scientific, Ghent, Belgium). Finally, the samples were washed twice in 1XPBS for 5’ each, mounted with Fluoromont (Sigma-Aldrich, Merck Chemicals, Overijse, Belgium), air-dried for 30’ with dehydration and sealed the cover slides with transparent nail polish.

Tumor vessel area was analysed by immunostaining CD31, which is a marker for endothelial cells, where the total CD31^+^ area was expressed as the percentage of the total tumor area. For analysis in QuPath, the entire image was annotated as a field (tumor tissue), where the number of cells were calculated using “cell detection” based on DAPI signal. Positive cell detection was then performed by setting threshold for the AF594 signal. The total number of endothelial cells then were determined as well as the total area of CD31^+^ positive cells compared to other cells. The relative level of CD31^+^ vessel density is then calculated as follows: ((CD31 area)/total area)*100 and expressed as percentage for all tissue sections and all tumors (please exemplary Figures M&M3, M&M4 for examples).

Tumor vessel maturation was assessed by the immunostaining for NG-2, which is a pericyte marker together with immunostaining for CD31. For analysis in QuPath, the entire image was annotated as a field (tumor tissue), where the number of cells were calculated using “cell detection” based on DAPI signal. Positive cell detection was then performed by setting threshold for the AF594 signal (CD31 positive) or AF488 signal (NG2 positive). For analysis, the CD31^+^ cells and NG2^+^ cells were artificially dilated twofold and any enlarged cell (typically the size of at least 2 cells), comprising both CD31 and NG2 signal is determined as a mature vessel. The total number of mature endothelial cells then were determined as well as the total area of NG2^+^CD31^+^ double positive cells compared to total number of CD31^+^ endothelial cells. The relative level of NG2^+^CD31^+^ vessel density is then calculated as follows: ((NG2^+^CD31^+^ area)/CD31^+^ area)*100 and expressed as percentage for all tissue sections and all tumors.

In addition, basement membrane deposition was assessed by immunostaining for laminin. The number of Laminin^+^ vessels was defined and is expressed as percentage total vessel area. For analysis in QuPath, this occurred similarly as for NG2 analysis, where here NG2 was replaced by laminin. The total number of mature endothelial cells then were determined as well as the total area of Lam^+^CD31^+^ double positive cells compared to total number of CD31^+^ endothelial cells. The relative level of Lam^+^CD31^+^ vessel density is then calculated as follows: ((Lam^+^CD31^+^ area)/CD31^+^ area)*100 and expressed as percentage for all tissue sections and all tumors (Please see exemplary Figure M&M4 as an example).

*Single cell analysis of NP uptake by image-based cytometry (ImageStream analysis)*

The tumor samples were dissociated into single cells using GentleMACS tissue dissociator and its kit (Miltenyi Biotec, Gladbach, Germany). The tumor samples were cut into small pieces, transferred in gentleMACS C-tubes containing RPMI/DMEM media and kit enzymes (tumor dissociation kit, Miltenyi Biotec, Gladbach, Germany) and were broken down using the gentleMACS. After the tumors were processed, the gentleMACS C-tubes were centrifuged 30’ on 1,500 rpm, the content of the gentleMACS C-tube was passed first through 70 um, followed by 40 um strainer and was centrifuged for 7’ on 300 g. The pellet was lysed using RBC lysis buffer for exactly 2’, centrifuged for 5’ on 300 g and resuspended in 1 mL media, which was after added slowly on top of 1 mL FBS to form a layer of media on top of the FBS. After centrifugation for 5’ on 100 g, the single tumor cells inside the media sank to the bottom of the FBS and the tumor cells were separated from the debris. The supernatant was removed, the cells were washed with 1XPBS, centrifuged for 5’ on 1.4 rpm and were incubated with Fc Blocker (1:100, in 1XPBS + 1%FBS, Thermo Fisher Scientific, Ghent, Belgium) for 30’ on ice. The cells were washed with 1XPBS + 1%FBS, centrifuged for 5’ on 1.4 rpm and incubated with two different antibody cocktails for 1 h on ice, protected from light, where all the antibodies were diluted in 1XPS + 1%FBS. The following antibodies were used in the first cocktail; anti-CD45 FITC (1:100, Thermo Fisher Scientific, Ghent, Belgium), anti-90.2 APC (2:100, Thermo Fisher Scientific, Ghent, Belgium), anti-CD3 AF610 (2:100, Thermo Fisher Scientific, Ghent, Belgium) and anti-CD144 PE (2:100, Thermo Fisher Scientific, Ghent, Belgium), and the following two antibodies in the second cocktail; anti-CD24 APC (3:100, Thermo Fisher Scientific, Ghent, Belgium) and anti-F4/80 FITC (3:100, Bio-Rad Laboratories, Temse, Belgium). The single cells were washed with 1XPBS + 1%FBS, resuspended in 1XPBS and transported in eppendorfs to the image-based cytometer Imagestream Mark II Imaging flow cytometer (Merck, Overijse, Belgium). Measurements were done by acquiring approximately 1 × 10^5^ single cells per sample. The images were acquired using the 60 × objective with the darkfield (780 nm laser) at 1 mW in order to reduce scatter light, while enabling darkfield-based detection of AuNPs inside cells. For the first cocktail, laser intensities were set at 1.00mW (488 nm), 20.00 mW (561 nm) and 50.00 mW (642 nm) and for the second cocktail, these were set at 1.00 mW (488 nm) and 100 mW (642 nm).

For analysis, iDEAS software (Amnis Corporation, USA) was used, followed by FCS Express 7.0 for visualization. First, focused and single cell were selected and gated, after which cell selections were gated based on the different markers used: tumor cells were defined as CD45-CD24+ , tumor-associated macrophages as F4/80+ , leukocytes as CD45+ , endothelial cells as CD144+ and cancer associated fibroblasts as CD45-CD90.2+ . For every cell type, darkfield images were taken and signal obtained in the darkfield channel were analysed for the different cell types, using bright intensity projection.

**Metabolomics**

E0771 cells were seeded in 6 well plates and exposed to Au or Au_Sil_ NMs at 50 µg/ml for 24h. Metabolites were then measured using spectrophotometric or fluorometric readouts using assay kits from Abcam for the different metabolites tested.

**Lipidomics**

E0771 cells (1*10^5^) were seeded in 6 well plates and exposed to Au or Au_Sil_ NMs at 50 µg/ml for 24h. The cells were collected by trypsinization and centrifugation, after which the pelleted cells were frozen at -80°C. The samples were then provided to LipoMetrix^TM^, where a targeted HILIC LC-MS/MS analysis was performed that includes +/-1800 lipid species. The results are presented as three independent replicates.

**Supporting Figures**

**Extended Data Figure S1.** Nanomaterial properties of the Au and Au_Sil_ particles used throughout this study, indicating the core diameter (in nm, measured by transmission electron microscopy (TEM)), hydrodynamic diameter (in nm, measured by dynamic light scattering (DLS)), polydispersity index (PdI) and the surface charge (in mV, expressed as the ζ-potential). Representative TEM micrographs are shown for the Au (left) and Au_Sil_ (right) NMs.

**Extended Data Figure S2. Distribution of Au and Au_Sil_ NMs in the TME upon intravenous administration.** The data are presented as violin plots for 4T1 tumors (orange) and E0771 tumors (black) for every parameter tested. Every violin plot represents a total of 16 animals per type of NM. a) The level of NMs taken up in the TME, as determined by ICP-MS and expressed as the relative % of injected dose. b) The tumor volume, as determined by caliper measurements. c) The area of necrotic tissue in the tumor as determined by H&E staining relative to the area of the entire tissue slice. d) The area positive for ECM in the tumor as determined by Picrosirius Red staining relative to the area of the entire tissue slice. e-i) Relative level of e) TAMs, f) CAFs, g) TECs, h) TILs and i) cancer cells (CC) expressed relative to the total number of cells as determined by ImageStreamX Mark II analysis. j) The relative area of tumor tissue taken up by blood vessels and k) the level of NG2^+^ and laminin-covered endothelial cells relative to the total number of endothelial cells. l-p) Relative % of l) TAMs, m) CAFs, n) TECs, o) TILs, p) CC positive for NMs as determined by ImageStreamX Mark II expressed relative to the total gated cell type. Significant differences between the groups are indicated where relevant (p < 0.05: *, p < 0.01: **) based on ANOVA testing using GraphPad 6.

**Extended Data Figure S3.** UMAP plots for each and every tumor-associated parameter determined (as displayed in Extended Data Figure S2) as a function of UMAP coordinates. For analysis, all data points for each and every animal were combined for animals receiving Au NPs. For every parameter, the values were first rescaled to a linear 0-1 scale with 0 being the lowest value for that parameter across all animals and 1 being the highest value for that parameter across all animals. The dots were colour-coded based on the upper left plot (the total tumor NP uptake level), where the 25% of animals with highest NP tumor levels were coloured red, the 25% of animals with lowest NP tumor levels were coloured blue and the remaining animals with medium NP levels were coloured green. To determine whether a particular parameter promotes or inhibits NP delivery efficacy, the red and blue groups should be separated on the Y-axis for the parameter.

**Extended Data Figure S4. Effects of Au and Au_Sil_ NM on isolated TECs.** a) Histograms showing the viability of Au and Au_Sil_ NM-treated TECs relative to that of untreated TECs. b-d) Graphs displaying the b) glycolysis level, c) glycolytic capacity and d) the glycolytic reserve based on the extracellular acidification rate (ECAR) of TECs when treated with Au or Au_Sil_ NMs. e) Histograms showing the relative uptake levels of Au (orange) and Au_Sil_ NMs (blue) in the absence of presence of endocytosis inhibitor PitStop2. The data are expressed relative to the values obtained for untreated control cells (= 100%). f,g) Volcano plots of genes involved in cellular motility and EMT transition signaling from TECs treated with f) Au or g) Au_Sil_ NMs compared to TECs that were left untreated. Genes that were significantly up- or downregulated are indicated by orange or blue colour codes, respectively. Control treatments groups received growth medium in the absence of NMs. Significant differences between the groups are indicated where relevant (****: p < 0.0001) based on ANOVA testing using GraphPad 6.

**Extended Data Figure S5. Effect of Au and Au_Sil_ NMs of CAF and TAMs.** a) Histogram revealing the permeability of TEC monolayers when exposed to conditioned media of NM-treated CAFs, relative to that of untreated TEC monolayers. b) Mobility of tumor cells exposed to conditioned media of NM-treated CAFs, relative to that of untreated tumor cells. c) Violin plots expressing the level of macrophage markers as determined by ImageStream analysis on primary isolated TAMs of 4T1 tumors, obtained from animals that were left untreated, or treated with Au or AuSil NMs. Additional controls were included to stimulate M1 phenotype (LPS + TNFα) or M2 phenotype (IL4 + IL13). d,e) Violin plots of d) M1 and e) M2 macrophage markers as determined by qPCR of primary TAMs isolated out of 4T1 tumors. NM-containing TAMs were further isolated by FACS sorting, using DyLight750 fluorophore-labeled NMs. qPCR was then performed only on the NM-containing TAMs. f) Histogram showing relative permeability of TEC monolayers as measured by fluorescently tagged dextran translocation when the TECs were exposed to supernatant of TAMs that were either left untreated, or had been exposed to Au or Au_Sil_ NMs. g,h) Relative migration of g) 4T1 and h) E0771 cells that had been exposed to the supernatants of TAMs that were either left untreated, or had been exposed to Au or Au_Sil_ NMs. i) Relative level of TAMs upon 1 day treatment with anti-CSFR1 antibody for 4T1 and E0771 tumors relative to untreated tumors. Control treatments groups received growth medium in the absence of NMs. Significant differences between the groups are indicated where relevant (*: p< 0.05; ****: p < 0.0001) based on ANOVA testing using GraphPad 6.

**Extended Data Figure S6. Effects of Au and AuSil on metastasis levels of TAM-depleted tumors.** a,b) The area taken up by metastatic nodules (a: 4T1; b: E0771) in the lung tissue slice of TAM-depleted tumors as determined by H&E staining relative to the area of the entire tissue slice. c,d) Expression of the truncated dLNGFR receptor that was introduced into the tumor cells and determined by qPCR of lung tissue in TAM-depleted tumors and expressed relative to the level of untreated tumor-bearing mice (c: 4T1; d: E0771). e,f) The level of Krt14 expression relative to that of housekeeping gene Hprt determined by qPCR of blood samples taken at 25 days post tumor grafting in TAM-depleted mice (e: 4T1; f: E0771). g,h) The number of EpCAM^+^ CD45^-^ cells detected in the blood at 25 days post tumor grafting in TAM-depleted mice expressed as the number of EpCAM^+^ cells per mL of sample and determined by ImageStream analysis (g: 4T1; h: E0771). i) The level of hypoxia as measured by in vivo optical imaging in tumor-bearing mice treated with Au or Au_Sil_ NM relative to the hypoxia level in untreated control animals (= 100%). Animals in the control group were administered PBS only. Significant differences between a treated group and untreated controls are indicated where relevant (p < 0.05: *) based on ANOVA testing using GraphPad 6 (*n* = 8).

**Extended Data Figure S7. Effects of *Redd1* KO in macrophages in NM-mediated tumor metastases.** a) Histograms displaying hematological parameters in WT -> WT and REDD1 KO -> WT chimeric mice, 5 weeks after bone marrow transplantation. Abbreviations: white blood cell (WBC), neutrophil (NEU), lymphocyte (LYM), monocyte (MON), eosinophil (EOS), basophil (BAS), red blood cell (RBC), platelet (PLT). b) Histogram expressing the tumor volume of E0771 tumors grafted in wild-type Bl6 mice or E0771 tumors grafted in animals having received *Redd1* KO bone marrow grafts. c) The number of metastatic nodules counted in the lungs for E0771 tumors at 25 days post grafting. d) The area taken up by metastatic nodules in the lung tissue slice as determined by H&E staining relative to the area of the entire tissue slice. e) Expression of the truncated dLNGFR receptor that was introduced into the tumor cells and determined by qPCR of lung tissue and expressed relative to the level of untreated tumor-bearing mice. f) The level of Krt14 expression relative to that of housekeeping gene Hprt determined by qPCR of blood samples taken at 25 days post tumor grafting. g) The number of EpCAM^+^ CD45^-^ cells detected in the blood at 25 days post tumor grafting expressed as the number of EpCAM^+^ cells per mL of sample and determined by ImageStream analysis. h,i) Histograms displaying immunohistochemical analysis of tumor tissues analysed for h) CD31^+^ vessel length or i) the level of NG2^+^ pericyte-covered CD31^+^ vessels. Data are expressed relative to the vessel length of pericyte coverage rate of untreated E0771 tumors grown in wild-type Bl6 mice. Animals in the control group were administered PBS only. Significant differences between the groups are indicated where relevant (p < 0.05: *; p < 0.01: **; p < 0.001: ***; p < 0.0001: ****) based on ANOVA testing using GraphPad 6.

**Extended Data Figure S8. Effects of Au and Au_Sil_ NM on tumor cells and related metastasis.** a,b) Relative ROS levels of a) 4T1 or b) E0771 cells treated with Au or Au_Sil_ NMs in the absence or presence of ROS scavenger NAC. c,d) Relative viability levels of c) 4T1 or d) E0771 cells treated with Au or Au_Sil_ NMs in the absence or presence of ROS scavenger NAC. e,f) Relative mitochondrial stress levels of e) 4T1 or f) E0771 cells treated with Au or Au_Sil_ NMs in the absence or presence of ROS scavenger NAC. g,h) Relative p62 levels, indicative of autophagic flux, for g) 4T1 or h) E0771 cells treated with Au or Au_Sil_ NMs or autophagy inducer rapamycin (RPM). i,j) Relative lysosomal pH levels for i) 4T1 or j) E0771 cells treated with Au or Au_Sil_ NMs or chlorpromazine (Chlor), which is known to induce lysosomal pH. k,l) Relative lysosomal pH levels for k) 4T1 or l) E0771 cells treated with Au or Au_Sil_ NMs. m,n) Relative lysosomal pH levels for m) 4T1 or n) E0771 cells treated with Au or Au_Sil_ NMs. o,p) Histograms revealing the significantly differentially expressed EMT-related genes for o) Au and p) Au_Sil_ NM treated cells. q) Representative photographs of different channels showing: 1) DAPI-stained nuclei (blue), 2) CD31-stained endothelial cells (green), 3) AF680-fluorescent Au NMs (magenta) or 4) merged image. The scale bars are 50 µm. The images at the bottom are magnifications of the rectangles indicated in the graph above. Control treatments groups received growth medium in the absence of NMs. Significant differences between the groups are indicated where relevant (p < 0.05: *; p < 0.01: **; p < 0.001: ***; p < 0.0001: ****) based on ANOVA testing using GraphPad 6.

**Extended Data Figure S9. Overview of Au and Au_Sil_ NM on lipid composition of NM-treated cells.** Every graph displays a colour-coded histogram displaying the relative composition of different lipids based on the degree of saturation and fatty acid chain length. Every graph shows 3 rows, with the top row depicting Au NM-treated cells, the middle row depicts Au_Sil_ NM-treated cells and the bottom row depicts untreated control cells. Abbreviations: FA: fatty acids, CE: cholesterol ester, CER: ceramide, DCER: dihydroceramide, DG: diacylglycerol, HexCer: hexosylceramide, LPC: lysophosphatidylcholine, LPE: lysophosphatidylethanolamine, PC: phosphatidylcholine, PC-O: Ether-linked phosphatidylcholines, PC-P: phosphate-linked phosphatidylcholines, PE: phosphatidylethanolamine, PE-O: Ether-linked phosphatidylethanolamine, PE-P: phosphate-linked phosphatidylethanolamine, PG: phosphatidylglycerol, PI: phosphatidylinositol, PS: phosphatidylserine, SM: sphingomyelin, TG: triacylglycerol.

**References**

1	Manshian, B. B. *et al.* High-content imaging and gene expression analysis to study cell-nanomaterial interactions: the effect of surface hydrophobicity. *Biomaterials* **35**, 9941-9950, doi:10.1016/j.biomaterials.2014.08.031 (2014).

2	Manshian, B. B., Munck, S., Agostinis, P., Himmelreich, U. & Soenen, S. J. High content analysis at single cell level identifies different cellular responses dependent on nanomaterial concentrations. *Sci Rep* **5**, 13890, doi:10.1038/srep13890 (2015).

3	Manshian, B. B., Pokhrel, S., Mädler, L. & Soenen, S. J. The impact of nanoparticle-driven lysosomal alkalinization on cellular functionality. *J Nanobiotechnology* **16**, 85, doi:10.1186/s12951-018-0413-7 (2018).

4	Izci, M. *et al.* The Efficacy of Nanoparticle Delivery to Hypoxic Solid Tumors by ciRGD Co-Administration Depends on Neuropilin-1 and Neutrophil Levels. *Advanced Healthcare Materials* **n/a**, 2300594, doi:<https://doi.org/10.1002/adhm.202300594>.

5	Maksoudian, C. *et al.* Gold Nanoparticle Delivery to Solid Tumors by Macrophage Depletion, Hypoxia Inhibition, and Collagen Degradation. *ACS Applied Nano Materials* **6**, 7605-7618, doi:10.1021/acsanm.3c00780 (2023).

6	Rossi, M. *et al.* PHGDH heterogeneity potentiates cancer cell dissemination and metastasis. *Nature* **605**, 747-753, doi:10.1038/s41586-022-04758-2 (2022).
